# Interleukin 15‐Presenting Nanovesicles with Doxorubicin‐Loaded Ferritin Cores for Cancer Immunochemotherapy

**DOI:** 10.1002/advs.202409194

**Published:** 2024-12-03

**Authors:** Yihui Zhai, Wen Zhang, Jinming Wang, Ying Kong, Rong Rong, Tianqun Lang, Chao Zheng, Yanke Wang, Yang Yu, Helen He Zhu, Ying Cai, Pengcheng Zhang, Yaping Li

**Affiliations:** ^1^ State Key Laboratory of Drug Research & Center of Pharmaceutics Shanghai Institute of Materia Medica Chinese Academy of Sciences 501 Haike Road Shanghai 201203 China; ^2^ University of Chinese Academy of Sciences Beijing 100049 China; ^3^ State Key Laboratory of Drug Research & Center of Pharmaceutics Shanghai Institute of Materia Medica Chinese Academy of Sciences Shanghai 201203 China; ^4^ China State Institute of Pharmaceutical Industry Shanghai 201203 China; ^5^ State Key Laboratory of Oncogenes and Related Genes Renji‐Med‐X Stem Cell Research Center Department of Urology Ren Ji Hospital School of Medicine and School of Biomedical Engineering Shanghai Jiao Tong University Shanghai 200127 China; ^6^ Yantai Institute of Materia Medica Shandong 264000 China; ^7^ National Facility for Protein Science in Shanghai Zhangjiang Lab Shanghai 201210 China; ^8^ Yantai Key Laboratory of Nanomedicine & Advanced Preparations Yantai Institute of Materia Medica Shandong 264000 China; ^9^ School of Biomedical Engineering & State Key Laboratory of Advanced Medical Materials and Devices ShanghaiTech University Shanghai 201210 China; ^10^ Shanghai Clinical Research and Trial Center Shanghai 201203 China

**Keywords:** CTLs, IL15, immunotherapy, nanovesicles, NK cells

## Abstract

Interleukin 15 (IL15) is crucial for fostering the survival and proliferation of nature killer (NK) cells and cytotoxic T lymphocytes (CTLs), playing a pivotal role in tumor control. However, IL15 supplementary therapy encounters challenges such as systemic inflammation and non‐specific stimulation of cancer cells. Herein, a nanovesicle termed DoxFILN, comprising a membrane presenting IL15/IL15 receptor α complexes (IL15c) and a core of doxorubicin‐loaded ferritin (Dox‐Fn) are reported. The DoxFILN significantly enhances the densities and activities of intratumoral CTLs and NK cells. Mechanistically, DoxFILN undergoes deshelling in the acidic tumor microenvironment, releasing Dox‐Fn and membrane‐bound IL15c. Dox‐Fn selectively target transferrin receptors on cancerous cells, facilitating intracellular Dox release and inducing immunogenic cell death. Concurrently, membrane‐bound IL15c recognizes and activates IL15 receptor β/γc heterodimers, leading to a remarkable increase in the proliferation and activation of CTLs (16‐fold and 28‐fold) and NK cells (37‐fold and 50‐fold). The IL15‐displaying nanovesicle introduced here holds promise as a potential platform for immunochemotherapy in the treatment of cancer.

## Graphical abstract

Nanovesicles with interleukin 15 (IL15) and IL15 receptor α complex (IL15c)‐presenting membrane and doxorubicin‐loaded ferritin (Dox‐Fn)‐based cores can disassemble in the tumor microenvironment to release Dox‐Fn and membrane‐bound IL15c, which targets cancer cells and effector cells, respectively, suppressing tumor growth in triple‐negative breast cancer mice models via facilitating infiltration and activity of effector cells of both innate and adaptive immunity.

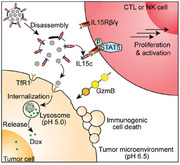

## 1 Introduction

Interleukin 15 (IL15) plays a pivotal role in disease control by fostering the survival and proliferation of effector cells from both innate and adaptive immunity.^[^ ^1^ ^]^ Elevated levels of intratumoral or peritumoral IL15 are positively correlated with a favorable prognosis in hepatocellular carcinoma or colorectal cancer patients.^[^ ^2^ ^]^ This correlation is grounded in IL15‐mediated activation, involving both neoantigen‐dependent and independent mechanisms, contributing to effective tumor eradication.^[^ ^3^ ^]^ Mechanistically, the anti‐tumor activity of IL15 relies on its ability to stimulate immune cells expressing IL15 receptor β/γc heterodimer (IL15Rβ/γc). This stimulation occurs through the membrane‐bound IL15/IL15 receptor α (IL15Rα) complex (IL15c), which is trans‐presented by various cells, particularly dendritic cells (DCs) exposed to inflammatory stimuli.^[^ ^4^ ^]^

Given the potent activity of IL15 in boosting anti‐tumor immunity, two types of IL15 supplementary therapy are undergoing clinical trials: one employing recombinant human IL15,^[^ ^5^ ^]^ and the other utilizing ALT‐803 (a superagonist complex of an IL15 mutein (N72D) bound to the sushi domain of IL15Rα fused to the immunoglobulin G1 Fc).^[^ ^6^ ^]^ While the successful expansion of natural killer (NK) cells and cytotoxic T lymphocytes (CTLs) has been realized, the effectiveness of these treatments faces limitations linked to the toxicity arising from elevated serum interferon‐γ (IFN‐γ) and interleukin 6 (IL6). This is likely attributed to non‐specific activation of immune cells. Additionally, IL15 has the potential to bind with IL15Rα on cancer cells, promoting their proliferation and migration independently of IL15Rβ/γc,^[^ ^7^ ^]^ thus counteracting its immunological anti‐tumor efficacy. To address these issues, IL15c‐expressing or ALT‐803‐loaded chimeric antigen receptor (CAR)‐modified T or NK cells have been explored.^[^ ^8^ ^]^ While significantly improving the tolerance of IL15‐based therapy, these strategies heavily rely on the specificity of CAR‐T and CAR‐NK cells and commonly suffer from on‐target off‐tumor adverse effects.^[^ ^9^ ^]^ Therefore, novel IL15 supplementary therapies are still of urgent need.

A prerequisite for successful IL15‐based treatments is the adequate infiltration of effector cells into the tumor. Therefore, combining treatments that can prime anti‐tumor immunity with IL15‐based therapy is crucial to achieve a synergistic impact, particularly for immune “cold” tumors. Notably, investigations into combining IL15‐based therapy with oncolytic viruses or radiotherapy have demonstrated synergistic therapeutic effects against various solid tumors.^[^ ^10^ ^]^ The ongoing clinical investigation (NCT02559674) involving the addition of ALT‐803 to standard chemotherapy underscores the potential benefits of such a combination. Cell‐specific delivery of the drugs to their corresponding target cells is crucial, as chemotherapeutic agents may inadvertently harm immune cells, compromising their biological functions, albeit inducing immunogenic cell death (ICD) of cancer cells.^[^ ^11^ ^]^ IL15 and serum soluble IL15Rα bound IL15 would lead to tumor progression via promoting cancer cell proliferation and migration and inducing systemic inflammation cytokines, respectively.^[^ ^12^ ^]^ To overcome these challenges, a meticulously tailored combination therapy approach is required.

**Figure 1:**
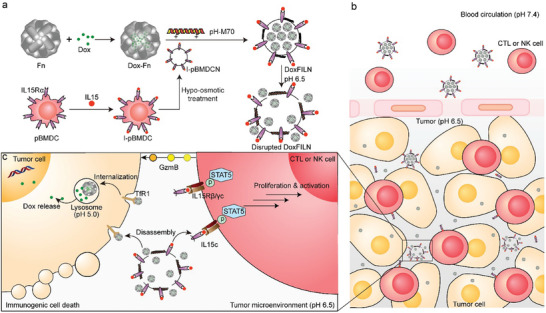
Schematic illustration of the preparation and mechanism of action of DoxFILN. a) Preparation of DoxFILN. LPS‐pretreated bone marrow‐derived dendritic cells (pBMDCs) were incubated with IL15 to obtain I‐pBMDCs. The I‐pBMDC membrane was isolated to give nanovesicles (I‐pBMDCN) for encapsulation of Dox‐Fn to obtain pH‐sensitive M70 peptide‐modified DoxFILN. b) After intravenous injection and tumor accumulation, DoxFILN was able to enhance the recruitment, proliferation, and activity of CTLs and NK cells in the tumor. c) DoxFILN was expected to disassemble into Dox‐Fn and I‐pBMDCN debris with the help of M70 under a mildly acidic tumor microenvironment. Dox‐Fn targeted TfR1 on cancer cells and released Dox in the lysosomes to induce ICD, while membrane‐bound IL15c recognized IL‐15Rβ/γc and thus facilitated the proliferation and activation of CTLs or NK cells through STAT5‐mediated pathway.

Triple‐negative breast cancer (TNBC) is the most aggressive type of breast cancer. However, the majority of TNBC patients have a poor prognosis even after combinational chemotherapy and immune checkpoint blockade, due to the low density and poor activity of tumor‐infiltrating lymphocytes (cold tumor).^[^ ^13^ ^]^ Upregulation of both IL15Rα and IL15 in TNBC cells is sufficient to activate immune cells through paracrine signaling, but it also induces the proliferation of cancer cells.^[^ ^14^ ^]^ Inspired by the fact that the biologically effective form of IL15 is membrane‐bound IL15c, we present a novel approach in the form of a pH‐responsive macrolittin 70 (pH‐M70)‐modified nanovesicle. This nanovesicle is composed of an IL15c‐presenting membrane and doxorubicin‐loaded ferritin (Dox‐Fn)‐based cores, named DoxFILN (**Figure** **1**a). Our hypothesis posits that DoxFILN can selectively accumulate and undergo deshelling in the acidic tumor microenvironment due to the activation of membrane disruptive peptide M70, leading to the release of Dox‐Fn and membrane‐bound IL15c (Figure 1b). While the Dox‐Fn component is designed to target transferrin receptor 1 (TfR1) on cancerous cells, releasing Dox in lysosomes to induce ICD, the membrane‐bound IL15c is expected to recognize IL‐15Rβ/γc, thereby facilitating the proliferation and activation of CTLs and NK cells through the signal transducers and activators of transcription 5 (STAT5)‐mediated pathway (Figure 1c). Using an orthotopic TNBC mice model, we demonstrated that DoxFILN increased the recruitment, proliferation, and activity of CTLs and NK cells, ultimately yielding remarkable anti‐tumor efficacy.

## 2 Results

**Figure 2:**
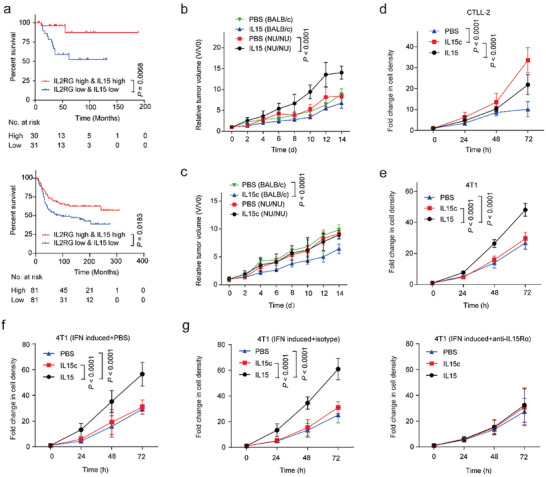
Association between intratumoral transcription of *IL15* and *IL2RG* and survival in TNBC patients and the effect of IL15 and IL15c on tumor growth and cell proliferation. a) Kaplan–Meier analysis of the overall survival (OS) probability of two cohorts TNBC patients (Sabatier: 83 patients; METABRIC: 216 patients) with different transcription levels of intratumoral *IL2RG* and *IL15* genes. Statistical analysis was calculated by a two‐sided log‐rank test. b,c) The tumor growth profiles of 4T1 tumor‐bearing mice following the indicated treatments. Treatments were initiated when the tumor volumes reached ≈100 mm^3^. IL15 (b) or IL15c (c) were dosed intratumorally (0.04 nmol per mouse at day 0, day 4, and day 8). Data were presented as mean ± s.d. (*n* = 6 biologically independent animals). Statistical significance was calculated by a two‐way ANOVA test. d,e) The growth profiles of CTLL‐2 (d) or 4T1 (e) cells following the indicated treatments in 72 h. 4T1 or CTLL‐2 cells (2 × 10^4^ cells) were incubated in 8 mL 1640 medium in the presence or absence of either IL15c or IL15 (0.2 nm). The number of live cells was determined using a cell counter after Trypan Blue (0.4%) staining. Data were presented as mean ± s.d. (*n* = 3 biological replicates per group). Statistical significance was calculated by a two‐way ANOVA test. f,g) 4T1 cells were pretreated by IFN‐γ (1 ng mL^−1^) for over 12 h. The growth profiles of 4T1 cells (2 × 10^4^ cells in 8 mL medium) after exposure to IL15 or IL15c (0.2 nm) at the presence of PBS (f) or antibodies (g). (*n* = 3 biologically independent samples).

Considering the pivotal role of IL15c‐IL15Rβ/γc interaction in IL15‐mediated anti‐tumor immunity, we evaluated the association between transcription of *IL15* (encodes IL15) and *IL2RG* (encodes IL15Rγc) genes with survival benefit in two cohorts of TNBC patients (Sabatier: 83 patients; METABRIC: 216 patients). The survival analysis revealed that patients with high intratumoral transcription of both *IL15* and *IL2RG* genes survived longer than those with low transcription of both genes in both cohorts (**Figure** **2**a). Conversely, elevated intratumoral transcription of either *IL15* or *IL2RG* alone did not demonstrate a survival benefit (Figure S1, Supporting Information). These results indicated that IL15 supplementary therapy might be more effective in treating tumors with sufficient infiltration of functional effector cells, which typically expressed IL15Rγc.

Based on this finding, we further evaluated the therapeutic effects of two types of IL15‐based supplementary therapies on both immunocompromised and immunocompetent mice bearing orthotopic 4T1 tumors. IL15 exhibited negligible benefits in immunocompetent mice and paradoxically accelerated tumor growth by 66% in immunocompromised counterparts (Figure 2b; Figure S2, Supporting Information). In contrast, IL15c significantly impeded tumor growth by 34% in immunocompetent mice while demonstrating no discernible effect in immunocompromised mice (Figure 2c; Figure S3, Supporting Information). To unravel the underlying mechanism, we monitored the proliferation of 4T1 and CTLL‐2 (murine CTL, a pivotal component of adaptive anti‐tumor immunity) in the presence or absence of supplementary IL15c or IL15. IL15c notably stimulated CTLL‐2 proliferation by threefold, proving to be 50% more effective than IL15 (Figure 2d). Conversely, IL15 accelerated 4T1 proliferation by 60% (Figure 2e). Given the presence of functional T lymphocytes in immunocompetent mice, in contrast to their absence in immunocompromised mice, our results underscored the pivotal role of IL15‐promoted CTL proliferation as a decisive factor in its anti‐tumoral activity. Furthermore, the disparate effects of IL15 and IL15c on 4T1 cell proliferation and tumor growth in immunocompromised mice hinted at the existence of a receptor on 4T1 cells selectively recognizing IL15 rather than IL15c.

The expression of IL15Rα on human basal‐like TNBC cell lines has been previously reported. We anticipated that 4T1 cells might also express IL15Rα, which could recognize IL15 but not IL15c. Since cancer cells in the tumors were exposed to interferon‐γ (IFN‐γ), we determined the expression of IL15Rα on 4T1 cells in the presence of different concentrations of IFN‐γ. IL15Rα was constitutionally expressed by 4T1 cells and could be significantly upregulated by IFN‐γ in a concentration‐dependent manner, reaching a 2.5‐fold increase (plateaued at 1 ng mL^−1^ IFN‐γ) (Figure S4, Supporting Information). Most of these receptors were ligand‐free and could form complexes with IL15. The IL15Rα on naïve 4T1 and IFN‐induced 4T1 cells could be saturated in the presence of 1 and 5 ng mL^−1^ IL15, respectively (Figure S5, Supporting Information). The level of IL15c on IFN‐γ‐induced cells was 2.2‐fold that of naïve cells, which was consistent with the ratio of IL15Rα between the two populations. Based on these findings, we further investigated whether the interaction between IL15 and IL15Rα on 4T1 cells could boost cell growth. Cell proliferation assay revealed that IL15 significantly promoted the proliferation of IFN‐γ‐pretreated 4T1 cells by twofold, while IL15c had no effect (Figure 2f). The activity of IL15 could be completely abolished by an anti‐IL15Rα antibody but not the isotype control (Figure 2g). These results clearly demonstrated that ligand‐free IL15Rα on 4T1 cells would bind IL15 in the tumor microenvironment, which could stimulate the proliferation of 4T1 cells.

Although IL15c was superior to IL15, intravenous injection of IL15 superagonist would cause systemic inflammation, probably due to its non‐specific accumulation.^[^ ^15^ ^]^ Inspired by the fact that native IL15c was of membrane‐bound form, we hypothesized that IL15c could be delivered using cell membrane‐derived nanovesicles. We previously found that nanovesicles derived from 4T1 cell membranes and myeloid cell membranes were able to target 4T1 tumors.^[^ ^16^ ^]^ To upregulate the expression of IL15Rα, bone marrow‐derived dendritic cells (BMDCs) and 4T1 cells were pretreated with lipopolysaccharide (LPS) and IFN‐γ, respectively. LPS‐pretreated BMDCs (pBMDCs) showed a threefold increase in membrane IL15Rα as well as an approximately onefold increase in co‐stimulation factor CD86 (Figure S6, Supporting Information). Western‐blotting results revealed that pBMDCs had higher IL15Rα expression than IFN‐γ‐treated 4T1 cells (Figure S7, Supporting Information). Most of the IL15Rα on pBMDCs were devoid of IL15 and could be saturated by incubating with IL15 (Figure S8, Supporting Information). The IL15‐incubated pBMDCs (I‐pBMDCs) were processed to generate I‐pBMDCs nanovesicles (I‐pBMDCNs). Both I‐pBMDCNs and IL15c showed comparable activity on CTLL‐2 and 4T1 cells, promoting phosphorylation of STAT5 (downstream of IL15Rβ/γc pathway) in CTLL‐2 to the same extent (Figure S9, Supporting Information). However, IL15c induced significant systemic inflammation after intravenous injection (Figure S10, Supporting Information). These results suggested that I‐pBMDCN could serve as an efficient and effective carrier for tumor‐targeted delivery of membrane‐bound IL15c.

**Figure 3:**
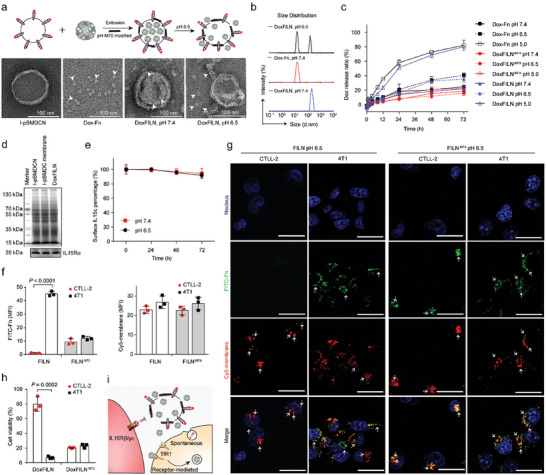
Preparation and characterization of DoxFILN and their interaction with 4T1 or CTLL‐2 cells. a) Representative schemes and transmission electron microscope (TEM) images of I‐pBMDCN, Dox‐Fn, and DoxFILN in different pH. The samples were stained with 2% phosphotungstic acid. b) Size distribution of DoxFILN and Dox‐Fn in buffers of pH 6.5 or pH 7.4. All data were determined by a Zetasizer. c) The Dox release profiles of Dox‐Fn, DoxFILN ^−M70^, and DoxFILN in buffers of pH 5.0, pH 6.4, or pH 7.4. All data were presented as mean ± s.d. (*n* = 3 samples per group). d) SDS‐PAGE protein analysis (top) and Western blot analysis of IL15Rα protein (bottom) in I‐pBMDCN, I‐pBMDC membrane, and DoxFILN. e) Profiles of the concentration of IL15c on DoxFILN in 72 h in pH 6.5 or pH 7.4. f) Flow cytometry determination of the cellular uptake of FITC‐labelled Fn and Cy5‐labelled membrane protein by 4T1 cells or CTLL‐2 cells after 4 h incubation with FILN^−M70^ or FILN in buffers of pH 6.5 (IL15c at 10 ng mL^−1^ equivalent). Data were presented as mean ± s.d. (*n* = 3 biological replicates per group) and statistically analyzed using the Student's *t*‐test. g) Representative confocal images showing intracellular localization of FITC‐labelled Fn and Cy5‐labelled membrane protein. The cells were stained with Hoechst 33 342 (blue). The scale bars represent a distance of 20 µm. h) Cell viability of 4T1 or CTLL‐2 after being treated by DoxFILN^−M70^ or DoxFILN in pH 6.5. Data were presented as mean ± s.d. (*n* = 3 biological replicates per group) and statistically analyzed using the Student's *t*‐test. i) Mechanism of cell‐specific drug delivery by DoxFILN.

A good infiltration of CTLs is a prerequisite for IL15c supplementary therapy. However, most human TNBC tumors and 4T1 tumors lack efficient intratumoral CTLs.^[^ ^15a^ ^]^ ICD can promote intratumoral CTL infiltration. Dox is a well‐proved inducer of ICD,^[^ ^17^ ^]^ which process involves the activation of caspase‐dependent apoptosis.^[^ ^18^ ^]^ Free Dox exerts cardiotoxicity,^[^ ^19^ ^]^ and nanosized formulations such as liposomal Dox (Doxil) has been developed to alleviate the toxicity. Given that many types of cancers, including TNBC, show elevated expression of TfR1,^[^ ^20^ ^]^ ferritin‐an endogenous protein that targets TfR1^[^ ^21^ ^]^ can be an ideal nanosized carrier for Dox. Therefore, TfR1‐targeting Dox‐Fn was chosen and prepared using a previously reported method.^[^ ^22^ ^]^ To create DoxFILNs, the Dox‐Fn were coated with I‐pBMDCN and further decorated with pH‐M70, which could resume its cell membrane rupture function under an acidic environment (Figures S11–S13, Supporting Information). The obtained DoxFILN contained an average of 48 ± 12 copies pH‐M70 molecules and 31 ± 16 Dox‐Fn. The typical loading capacity and encapsulation efficiency of Dox were 3.1 ± 1.0% and 92.1 ± 5.3%, respectively. The core–shell‐structured DoxFILN (pH 7.4) could rupture to release the encapsulated Fn under pH 6.5 (**Figure** **3**a; Figure S14, Supporting Information). Accordingly, dynamic light scattering results revealed the emergence of a new population of nanoparticles (≈10 nm in diameter) after incubating DoxFILN (≈125 nm in diameter under pH 7.4) in the buffer of pH 6.5 (Figure 3b; Figure S15, Supporting Information). In contrast, DoxFILN remained stable for 8 h at 37 °C in buffer (pH 7.4) under shaking conditions (Figure S16, Supporting Information). The release of Dox from DoxFILN was also pH‐ and M70‐dependent and efficient Dox release (>80%) was only observed when DoxFILN or Dox‐Fn were incubated in a buffer of pH 5.0 (Figure 3c). The membrane‐bound protein such as IL15c, retained stable during the preparation of DoxFILN and was stable for 72 h at both pH 7.4 and pH 6.5 (Figure 3d,e). These results revealed an M70‐mediated and pH‐triggered stepwise release of Dox‐Fn and Dox from DoxFILN and Dox‐Fn, respectively. The stable retention of Dox in Dox‐Fn at pH 6.5 ensured TfR1‐mediated delivery of Dox to cancer cells in an acidic tumor microenvironment.

Given the separation of Fn and membrane‐bound IL15c at pH 6.5, we aimed to monitor their fates in a co‐culture system containing both 4T1 and CTLL‐2 cells under pH 6.5 using flow cytometry (Figure S17, Supporting Information). The expressions of TfR1 and IL15Rγc on 4T1 cells and CTLL‐2 cells were first determined. CTLL‐2 cells showed higher IL15Rγc expression but lower TfR1 expression compared to 4T1 cells (Figures S18,S19, Supporting Information). For FILN (a Dox‐free version of DoxFILN)‐treated cells, Fn was almost exclusively found in 4T1 cells (40‐fold of that in CTLL‐2), while the uptake of membrane‐bound IL15c was comparable in the two cell lines (Figure 3f; Figure S20, Supporting Information). No cell‐specific endocytosis of either Fn or membrane‐bound IL15c was observed in FILN^−M70^ (a pH‐insensitive FILN by removing M70)‐treated cells. This result was further confirmed by confocal microscopy (Figure 3g). The images showed that Fn and membrane‐bound IL15c were poorly colocalized in FILN‐treated CTLL‐2 cells but largely colocalized in FILN^−M70^‐treated CTLL‐2 and 4T1 cells. The selective uptake of Fn by 4T1 cells enabled preferential delivery of Dox to the cancer cells by DoxFILN compared with Dox‐I‐pBMDCN (I‐pBMDCN loaded with free Dox) (Figure S21, Supporting Information). As a result, DoxFILN preferentially killed 4T1 cells with little effect on CTLL‐2 cells, while DoxFILN^−M70^ non‐specifically killed both cells (Figure 3h). These results demonstrated that DoxFILN ruptured in a mildly acidic environment, liberating Dox‐Fn from membrane‐bound IL15c (Figure 3i). The released Dox‐Fn could recognize and deliver Dox into TfR1‐overexpressing cancer cells, while membrane‐bound IL15c would specifically bind to IL15Rβ/γc on CTLL‐2 cells or be non‐specifically phagocytosed by 4T1 cells.

**Figure 4:**
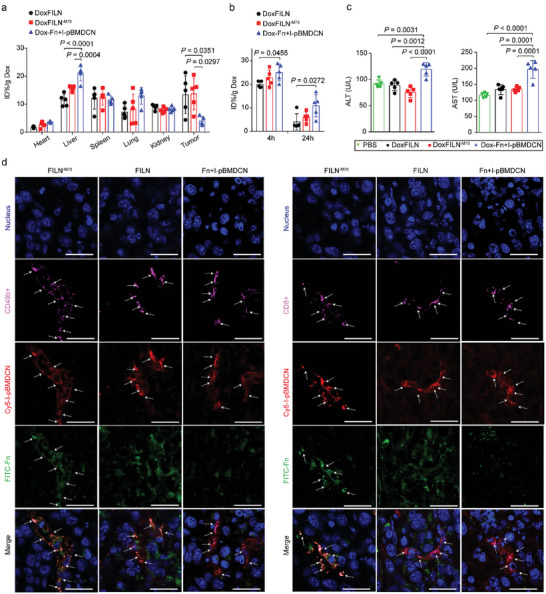
Accumulation of DoxFILN in the tumors. a) The distribution of Dox in the major organs and tumors at 8 h after injection (Dox: 5 mg kg^−1^). Data were presented as mean ± s.e.m. (*n* = 5 biologically independent animals). b) The distribution of Dox in the livers at 4 and 24 h after injection. Data were presented as mean ± s.e.m. (*n* = 5 biologically independent animals). c) Serum ALT and AST after 5 days of one injection of DoxFILN, DoxFILN^−M70^ and Dox‐Fn + I‐pBMDCN at 40 mg kg^−1^ Dox equivalents Data were presented as mean ± s.d. (*n *= 5 biologically independent animals). d) Representative confocal images showing localization of FITC‐labelled Fn and Cy5‐labelled membrane protein. The cells were stained with DAPI (blue), CD49b (pink), or CD8 (pink). The scale bars represent a distance of 25 µm. Statistical significance was analyzed using one‐way ANOVA and Tukey's test.

We then investigated the efficiency of DoxFILN in tumor‐targeted delivery of Dox and membrane‐bound proteins such as IL15c using liquid chromatography‐mass spectrometry (LC‐MS) and IVIS Spectrum Imaging System (PerkinElmer, *λ*_Ex_/*λ*_Em_ = 650/670 nm), respectively. Tumor accumulation of Dox and membrane‐bound IL15c peaked at 8 h after intravenous injection of DoxFILN or DoxFILN^−M70^ (Figure S22, Supporting Information). Fast tumor accumulation and clearance of Dox from the tumors were observed in mice receiving a mixture of Dox‐Fn and I‐pBMDCN. Further pharmacokinetics results confirmed that DoxFILN prolonged the circulation of Dox‐Fn (Figure S23, Supporting Information). At 8 h after the injection, the tumor accumulation of Dox in DoxFILN‐ and DoxFILN^−M70^‐treated mice was 2.4‐fold higher than that in mice receiving the mixture (**Figure** **4**a). In addition to a lower intratumoral deposition, free Dox‐Fn in the mixture showed a higher liver accumulation (Figure 4a,b), potentially causing liver tissue damage at high dosage (40 mg kg^−1^ Dox) evidenced by elevated serum alanine aminotransferase (ALT) and aspartate aminotransferase (AST) and increased myeloid cell infiltration in the liver compared to PBS group (Figure 4c; Figures S24,S25, Supporting Information). The intratumoral distribution of Fn and membrane‐bound IL15c was further studied using confocal microscopy. Among the three treatments, Fn in the Fn + IpBMDCN group exhibited lower tumor accumulation (Figure 4d; Figure S26, Supporting Information). Membrane‐bound IL15c largely colocalized with NK (CD49b^+^) and CTL (CD8^+^) cells in tumors collected from all the groups, while strong colocalization of Fn with membrane‐bound IL15c was only observed in the tumors treated with FILN^−M70^ (Figure 4d). These results demonstrated that I‐pBMDCN encapsulation of Dox‐Fn was crucial for improving tumor accumulation and reducing the potential hepatotoxicity of Dox‐Fn. The results also suggested that the pH‐responsive release of Dox‐Fn in the acidic tumor microenvironment could decrease Dox accumulation within or near NK cells and CTLs while facilitating their intratumoral accumulation.

**Figure 5:**
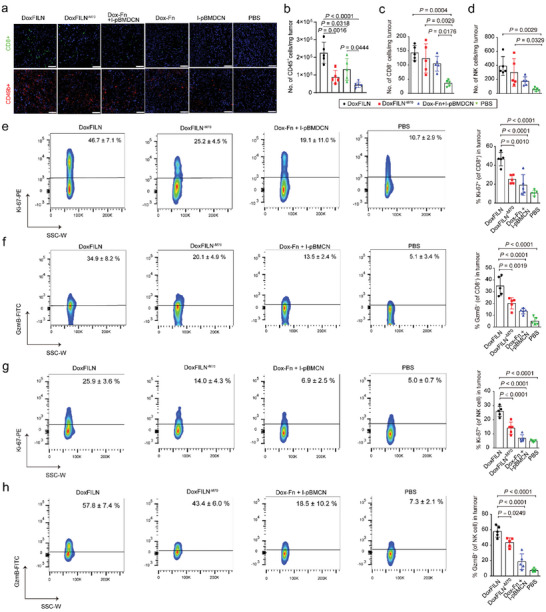
Effect of DoxFILN on anti‐tumor immunity. a) Representative fluorescence images of tumor slices that were collected from mice receiving indicated treatments and were analyzed with CaseViewer version 2.0 (3DHistech, Hungary) (Dox: 5 mg kg^−1^; IL15c: 85 pmole per mouse, 3 injections, 1 injection every 4 days). CD8 (green) and CD49b (red) were visualized using fluorescence‐labelled antibodies. The nucleus was stained with DAPI (blue). The scale bars represent a distance of 50 µm. b–d) Densities of intratumoral immune cells (b) CD8^+^ T cells (c) and NK cells (d). e–h) Percentages of proliferative (Ki‐67^+^) and active (GzmB^+^) cells among CD8^+^ T cells (e,f) and NK cells (g,h). All the animals were analyzed 7 days after the last injection of the 3‐injection regimen (Dox: 5 mg kg^−1^; IL15c: 85 pmole per mouse, 3 injections, 1 injection every 4 days). All the animals were analyzed 7 days after the last injection of the 3‐injection regimen (Dox: 5 mg kg^−1^; IL15c: 85 pmole per mouse, 3 injections, 1 injection every 4 days). All data were presented as mean ± s.d. (*n* = 5 biologically independent animals). Statistical significance was analyzed using one‐way ANOVA and Tukey's test.

Given the potential of DoxFILN in tumor‐targeted and cell‐specific delivery of Dox‐Fn and membrane‐bound IL15c, we measured the efficacy of DoxFILN in priming anti‐tumor immunity. We first investigated the effects of treatments on DC maturation in the draining lymph nodes (DLNs) and the density of effector cells in the tumors using flow cytometry. DoxFILN was the most effective in promoting DC maturation (CD80^+^CD86^+^), and the ratio of mature DC from DoxFILN‐treated mice was 3.4‐fold, 1.7‐fold, and 1.4‐fold of those from PBS‐treated, mixture‐treated, and DoxFILN^−M70^‐treated ones, respectively (Figure S27a, Supporting Information). This trend was positively correlated with the differences in their abilities to induce ICD both in vitro and in vivo (Figure S28, Supporting Information). Further ex vivo co‐stimulation assay confirmed that the DCs harvested from the DLNs of DoxFILN‐treated B16F10‐OVA tumor‐bearing mice were more potent in stimulating the proliferation of OT I cells compared to those from mice receiving the rest treatments (Figure S29, Supporting Information). A similar trend was observed in the intratumoral densities of CD8^+^ T cells and NK cells (Figure S27b,c, Supporting Information), which was further confirmed by confocal images of tumor tissues after the same treatments (**Figure** **5**a). Dox‐Fn or I‐pBMDCN alone showed limited efficacy compared to PBS. All the treatments did not change the density of intratumoral T_Reg_ (Figure S27d, Supporting Information). These results demonstrated that combinational immunochemotherapy was beneficial, with DoxFILN being the most effective in vivo.

We then dedicated to understand how the form of combination therapy could influence the activity of effector cells in both innate and adaptive immunity, as inappropriate chemotherapy had been reported to inhibit anti‐tumor immunity by damaging lymphocytes and myeloid cells.^[^ ^11^, ^23^ ^]^ Flow cytometry analysis showed that the tested treatments did not cause a decrease in intratumoral infiltration of immune cells (Figure 5b). In addition, all the combinational treatments increased the intratumoral density of CTLs compared to the PBS group (Figure 5c). However, more proliferative (Ki‐67^+^) and active (GzmB^+^) CTLs were observed in the DoxFILN‐treated tumors than those in the DoxFILN^−M70^‐ or mixture (Dox‐Fn + I‐pBMDCNs)‐treated tumors (Figure 5e,f; Figure S30, Supporting Information). The intratumoral densities of proliferative and active CTLs in DoxFILN‐treated tumors were 16‐fold and 28‐fold higher than those in PBS‐treated tumors. We further assessed the intratumoral recruitment of antigen‐specific CTL in mice bearing B16F10‐OVA tumors. The intratumoral density of OVA_257–264_‐H‐2Kb‐specific CTLs in DoxFILN‐treated mice was 19‐fold higher than that in mice receiving PBS (Figure S31, Supporting Information). Similarly, the DoxFILN was more potent than the DoxFILN^−M70^ and the mixture in increasing the percentages of proliferative and active NK cells (Figure 5d,g,h; Figure S32, Supporting Information). The intratumoral densities of proliferative and active NK cells in DoxFILN‐treated tumors were 37‐fold and 50‐fold higher than those in PBS‐treated tumors. These results demonstrated that simultaneous transportation and subsequent dissociation of Dox‐Fn and membrane‐bound IL15c were two critical properties leading to the supreme efficacy of DoxFILN.

**Figure 6:**
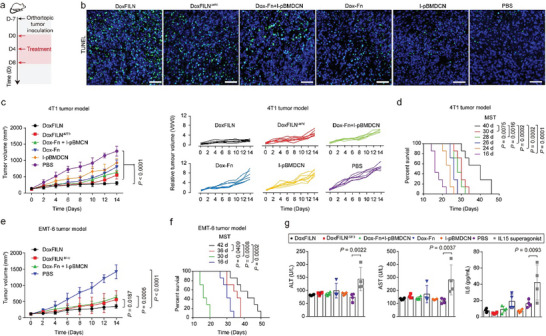
Anti‐tumor efficacy and biocompatibility of DoxFILN in vivo. a) Experimental timeline and groups (3 intravenous injections, Dox: 5 mg kg^−1^; IL15c: 85 pmole per mouse). b) Fluorescence images of tumor tissues stained with TUNEL at day 14. The scale bars represent a distance of 50 µm. c,d) Growth profiles of 4T1 tumors (c) and survival curves (d) of mice receiving the indicated treatments (Dox: 5 mg kg^−1^; IL15c: 85 pmole per mouse, *n* = 7 biologically independent animals). e,f) Growth profiles of EMT‐6 tumors (e) and survival curves (f) of mice receiving the indicated treatments (Dox: 5 mg kg^−1^; IL15c: 85 pmole per mouse, *n *= 7 biologically independent animals). Statistical analyses were performed by using two‐way ANOVA and Tukey's tests for tumor growth data and log‐rank tests for survival data, respectively. g) Liver enzymes (ALT, AST) and serum cytokine (IL6) were measured from serum collected on day 3 after the last injection. Data were presented as mean ± s.d. (*n* = 4 biologically independent animals).

Given the potent activity of DoxFILN in activating innate and adaptive immune response, we then evaluated the efficacy of DoxFILN in vivo through a 3‐injection treatment regimen (1 injection every 4 days) (**Figure** **6**a). Terminal deoxynucleotidyl transferase dUTP nick end labeling (TUNEL) staining results qualitatively showed that DoxFILN treatment resulted in elevated apoptosis in tumors, which was much higher than that observed with the rest treatments, thereby confirming a synergistic anti‐cancer efficacy (Figure 6b; Figure S33, Supporting Information). In an orthotopic 4T1 tumor model, the tumor growth was greatly retarded by DoxFILN (75% growth inhibition), while DoxFILN^−M70^ and Dox‐Fn + I‐pBMDCN with the same regimen showed 48% and 42% growth inhibition 14 days after the first injection. Dox‐Fn or I‐pBMDCN alone inhibited 29% and 22% of tumor growth during the same period (Figure 6c; Figure S34, Supporting Information). Meanwhile, there was no significant change in the body weight of mice during treatment (Figure S35, Supporting Information). Besides, the MST of mice was extended from 16 days (PBS group) to 40, 30, 28, 26, and 24 days after the treatments with DoxFILN, DoxFILN^−M70^, Dox‐Fn + I‐pBMDCN, Dox‐Fn, and I‐pBMDCN, respectively (Figure 6d). In another murine TNBC model (EMT‐6), which also had high expression of TfR1 as 4T1 model (Figure S36, Supporting Information), the tumor growth was inhibited by 77% after DoxFILN treatment (Figure 6e), and the MST of mice was extended from 16 days (PBS group) to 42 days (Figure 6f), confirming that DoxFILN was a potential treatment for TNBC. Further study confirmed that DoxFILN was the most efficient in controlling tumor mass without causing a noticeable decrease in body weight or pathological change in major organs, while free Dox caused cardiotoxicity (Figure S37, Supporting Information). These treatments did not increase serum ALT, AST, creatinine (CREA) and UREA, or cytokines (IL6 and IL10) under the same treatment regimen (Figure 6g; Figure S38, Supporting Information). On the contrary, mice treated with free IL15 superagonist at the same dosage showed elevated serum AST, ALT, and IL6, indicating the occurrence of hepatotoxicity. The reduced toxicity of DoxFILN may be associated with its shorter half‐life compared to the IL15 superagonist (Figure S39, Supporting Information). These results demonstrated that DoxFILN was an effective and safe treatment for TNBC.

## 3 Discussion

The majority of TNBC patients respond poorly to chemotherapy and immunotherapy due to the low density and activity of tumor‐infiltrating lymphocytes.^[^ ^24^ ^]^ Current IL15‐based supplementary therapy (recombinant human IL15 and IL15 superagonist) can stimulate intratumoral NK cells and CTLs by paracrine pathway but is limited by the potential risk of promoting IL15Rα‐mediated cancer cell proliferation and systemic inflammation.^[^ ^8^, ^14^ ^]^ Inspired by the fact that membrane‐bound IL15c is the native activator of high‐affinity IL15Rβ/γc‐expressing cells,^[^ ^25^ ^]^ we postulated that an IL15c‐overexpressing bone marrow‐derived dendritic cell (BMDC) membrane‐derived nanovesicle could function as a tumor‐targeted IL15β/γc agonist and serve as a carrier for Dox‐Fn, a tumor cell‐targeted immunogenic cell death (ICD) inducer.^[^ ^26^ ^]^ In this study, we have successfully demonstrated the development of a pH‐responsive nanovesicle, named DoxFILN, composed of an IL15c‐presenting membrane and a Dox‐Fn‐based core. This nanovesicle significantly enhances the intratumoral densities and activities of both NK cells and CTLs, presenting a promising approach for effective immunochemotherapy of TNBC.

The design of our DoxFILN is based on our finding that only TNBC patients with high intratumoral transcription of both *IL15* and *IL2RG* have a favorable prognosis than those with low transcription of the two genes, which indicates that effective anti‐tumor immune surveillance requires the infiltration and activation of effector cells. Our DoxFILN has three unique features. First, membrane‐bound IL15c is employed as an activator of IL15Rβ/γc‐expressing effector cells. Membrane‐bound IL15c is a native form of IL15 that binds with IL15Rβ/γc with higher affinity than its soluble forms.^[^ ^25^ ^]^ In addition, the multivalent presentation of membrane‐bound IL15c on the nanovesicles is crucial for inducing optimal immune responses.^[^ ^27^ ^]^ Second, Dox‐Fn is used for cancer cell‐specific delivery of Dox. ICD inducers such as Dox can lead to lymphodepletion due to their non‐specific accumulation in the immune cells.^[^ ^23^ ^]^ Dox‐Fn can preferentially accumulate in 4T1 cells through TfR1, the expression of which is much higher on 4T1 cells than that on immune cells. However, TfR1 is also widely expressed in normal organs, albeit at relatively lower levels. Thus, the third key feature of our design is that Dox‐Fn is transported within pH‐responsible and IL15c‐presenting nanovesicles, which have shown tumor‐targeting capability.^[^ ^16^, ^28^ ^]^ The Dox‐Fn is designed to be released in the tumor microenvironment with the help of pH‐M70 to facilitate its tumor penetration and TfR1‐mediated endocytosis by cancer cells. As a result, the membrane‐bound IL15c and Dox‐Fn could accumulate in the tumors as a whole while modulating their own target cells, thereby inducing sufficient infiltration, proliferation, and activation of the effector cells.

## 4 Conclusion

We have demonstrated a core–shell structured nanovesicle, DoxFILN, for tumor‐targeted delivery of membrane‐bound IL15c and Dox. And we developed for the first time a membrane‐bound form of IL15c for tumor‐specific effector cell activation. In the tumor, the two components could dissociate and interact specifically with their own target cells via ligand‐receptor recognition‐mediated pathways. The cell‐specific delivery of the two drugs induces ICD in cancer cells and promotes the proliferation of effector cells without off‐target side effects, such as effector cell depletion or cancer cell proliferation activation. We envision that the described strategy of delivering two drugs targeting different intratumoral cells is of critical importance for the modulation of multiple cell types within the tumor, ultimately enhancing cancer immunotherapy.

## 5 Experimental Section

### Materials

Doxorubicin and 2,3‐dimethylmaleic anhydride were purchased from Coupling Medical Technology Co., Ltd (Shanghai, China). Macrolittin 70 (M70, NH_2_‐GIGEVLKELATLLPELQSWIKAAQQL‐OH, >98%) was custom synthesized by Top‐peptide Co., Ltd. (Shanghai, China). 2‐hydroxyethyl disulfide was purchased from Alfa Aesar (Beijing, China). Ferritin was purchased from Merck Co., Ltd. (Darmstadt, Germany). Trypsin, fetal bovine serum (FBS), IL4, GM‐CSF, IL2, RPMI 1640, Dulbecco's modified Eagle medium (DMEM), and PBS were obtained from ThermoFisher (Shanghai, China). Antibiotics, DAPI, and Hoechst 33 342 were purchased from Yeasen (Shanghai, China). BSA, FITC, and Cy5 were purchased from Meilun Biotechnology Co., Ltd. (Dalian, China). Protease Inhibitor Cocktail was obtained from Selleck Chem (Houston, USA). MACS LS column was purchased from Miltenyi Biotech. Carboxyfluorescein succinimidyl ester (CFSE) was obtained from Sigma. ELISA kits for IL15c, IL6, IL10, IFN‐γ, and TNF‐α assay were all purchased from Neobioscience Co., Ltd. (Shenzhen, China) and the high‐mobility group box 1 (HMGB1) ELISA kit was purchased from Solarbio Science & Technology Co., Ltd. (Beijing, China). Other chemicals were of analytical grade and obtained from Sinopharm Chemical Reagent Co., Ltd. (Shanghai, China) unless otherwise indicated. Ultrapure water was produced with a Milli‐Q water purification system.

### Cell Culture

The murine TNBC cell line 4T1 and EMT6 and the melanoma cell line B16F10‐OVA were purchased from the Cell Bank of Shanghai, Chinese Academy of Sciences (Shanghai, China). The 4T1 and EMT6 cells were cultured in RPMI 1640 containing 10% FBS, 2.5 g L^−1^ glucose, 0.11 g L^−1^ sodium pyruvate, and 1% antibiotics (complete 1640 medium). The melanoma cell line B16F10‐OVA was maintained in DMEM supplemented with 10% FBS and 1% antibiotics (penicillin/streptomycin). Murine cytotoxic T cell line CTLL‐2 was originally sourced from the American Type Culture Collection, and cultured in a complete 1640 medium with 100 U mL^−1^ IL2. All the cells were maintained at 37 °C in a humidified and 5% CO_2_ incubator.

### Animals

Female BALB/c mice (20–22 g) and female C57BL/6 mice (20–22 g) were purchased from Shanghai Experimental Animal Center. Female OT I mice (20–22 g) were received from the Shanghai Institute of Immunology. Mice bearing orthotopic 4T1 and EMT6 tumors were established by injecting 100 µL of cancer cell suspension (1 × 10^6^ cells) to the second right mammary gland of female BALB/c mice and were used for in vivo study when the size of the tumor reached ≈100 mm^3^. For B16F10‐OVA tumor models, 1 × 10^6^ B16F10‐OVA tumor cells (in 100 µL) were injected into the right flank of C57BL/6 mice. All animal procedures were performed under the guidelines approved by the Institutional Animal Care and Use Committee of the Shanghai Institute of Materia Medica, Chinese Academy of Sciences.

### Antibodies

The anti‐IL15Rα, anti‐IgG isotype antibody used for in vitro efficacy study was purchased from R&D (cat. no. AF551; cat. no. AB‐108‐C). The fluorescence‐labelled primary antibodies used for immunostaining were against mouse CD45 (ebioscience, cat. no. 56‐0451‐82), mouse CD3 (eBioscience, cat. no. 45‐0031‐82), mouse CD4 (eBioscience, cat. no. 11‐0042‐82), mouse CD8 (eBioscience, cat. no. 17‐0081‐81), mouse CD49b (eBioscience, cat. no. 17‐5971‐81), mouse MHC‐II (biolegend, cat. no. 107 625), mouse CD11c (eBioscience, cat. no. 11‐0114‐81), mouse CD80 (eBioscience, cat. no. 12‐0801‐81), mouse CD86 (eBioscience, cat. no. 17‐0862‐81), mouse intracellular Ki‐67 (eBioscience, cat. no. 12‐5698‐80), mouse intracellular Granzyme B (eBioscience, cat. no. 11‐8898‐80), mouse intracellular Foxp3 (eBioscience, cat. no. 12‐5773‐80), T‐Select H‐2Kb OVA_257‐264_ Tetramer (MBL Life Science, cat. no. TS‐5001‐1C). The stained cells were analyzed on BD Fortessa or FACS Calibur flow cytometers (USA) and with the FlowJo software package (version 10.6.2; TreeStar, USA). The primary antibodies used for cell sorting were CD8a (biotinylated anti‐mouse CD8a antibody, Biolegend, cat. no. 117 304) and CD11c (biotinylated anti‐mouse CD11c antibody, Biolegend, cat. no. 100 704). The primary antibodies used for Western blot were against IL15Rα (R&D, AF551), IL2RG (proteintech, 11409‐1‐AP), Transferrin Receptor (abcam, cat. no. ab214039), Phospho‐Jak1 (Cell Signaling Technology, 74129S), Phospho‐Stat5 (Cell Signaling Technology, 4322T). The secondary antibodies used for Western blot were horseradish peroxidase‐labeled anti‐rabbit IgG (H+L) (Simuwubio, cat. no. SD0039), horseradish peroxidase‐labeled anti‐goat IgG (H+L) (Simuwubio, cat. no. SD0040). The primary antibodies used for immunofluorescence staining were against CD8 (Servicebio, cat. no. GB11068), CD49b (abcam, ab181548), CRT (Affinity Biosciences, cat. No. DF3139), HMGB1 (Affinity Biosciences cat. No. AF7020) The secondary antibodies used for immunofluorescence staining were Cy3‐labelled goat anti‐rabbit secondary antibody (Servicebio, cat. no. GB21303), Alexa Fluor 488‐labelled goat anti‐rabbit secondary antibody (Servicebio, cat. no. GB25303), Alexa Fluor 488‐labelled rabbit anti‐mouse secondary antibody (Yeasen, cat. no. 33906ES60), Alexa Fluor 647‐labelled rabbit anti‐mouse secondary antibody (Yeasen, cat. no. 33913ES60).

### Bioinformatic Analysis

Gene expression data were derived from non‐repeated samples of breast‐invasive carcinoma patients in the METABRIC dataset (*n* = 2509) and Sabatier et al. data (*n* = 266) that were acquired from the cBioPortal database (www.cbioportal.org) and GEO dataset (GSE21653). TNBC patients were manually selected based on the immunohistochemistry findings. Patients with other causes of death (for the METABRIC dataset) or without gene expression data were excluded from further analysis, 216 (METABRIC dataset) and 83 (GSE21653) TNBC patient samples were subsequently included in this study. Survival curves were calculated and drafted in R (3.6.1) using the survminer (0.4.6) and survival (2.44‐1.1) packages.

### Preparation of I‐pBMDCN

I‐pBMDCN was freshly prepared from pBMDC. To obtain pBMDC, the bone marrow was obtained by flushing the femur and tibia with RPMI 1640 medium containing 10% FBS. The obtained cells were then cultured in a medium containing 20 ng ml^−1^ GM‐CSF and 10 ng mL^−1^ IL4. On days 2, 4, and 6, half of the culture supernatant was removed, and an equal volume medium containing 20 ng ml^−1^ GM‐CSF and 10 ng mL^−1^ IL4 was added to the plates. On day 7, lipopolysaccharide (LPS, Sigma) was added into the medium at 1 µg ml^−1^. On day 8, pBMDC was induced. I‐pBMDC was an IL15Rα saturated form of pBMDC which obtained by incubated with IL15 (25 ng mL^−1^) for more than 30 min.

I‐pBMDC was then disrupted in cold Tris buffer (pH 7.4, with 1 × EDTA‐free protease inhibitors) for 1 h at 4 °C and further centrifuged at 500 g for 10 min. The supernatants were sonicated and centrifuged at 10^4^ × g for 10 min (Biofuge Stratos, Thermo). The supernatants were further centrifuged at 10^5^ × g for 1 h (Himac CS GXII, Hitachi). The resulting cell membrane pellets were resuspended with 10 mm Tris buffer and sonicated for 30 s using an ultrasonicator (JYD‐650L, Zhixin Inc., China) before further extruding sequentially through 200 and 100 nm polycarbonate membranes with LF‐1 (Avastin, Canada) to give I‐pBMDCN.

### Preparation of Dox‐Fn Cores

The Dox‐Fn cores were prepared according to a previously reported method.^[^ ^22^ ^]^ Briefly, Ferritin was dissolved in 8 m urea (Sinopharm Chemical Reagent Co., Ltd.) at a concentration of at 1 mg mL^−1^. After a 30 min gentle vortex at room temperature, the Dox was added into the solution with a final concentration of 1 mg mL^−1^. After 10 min incubation in the dark, the mixture was dialyzed using dialysis bags (molecular weight cut‐off 3500 Da) against gradient concentrations of urea buffer (7, 5, 3, 2, 1, and 0 m, each for 4 h) containing 1 mg mL^−1^ of Dox at 4 °C. The resulting solution was then dialyzed against saline overnight to remove the free Dox.

### Preparation of DoxFILN

Dox‐Fn cores (10 mg mL^−1^, 1 mL, pH 7.4) were mixed with I‐pBMDCN (derived from 10^8^ cells, 500 µL), followed by a 2 min sonication (JYD‐650L, Zhixin Inc., China). The prepared nanovesicles were further incubated with 35 µL pH‐M70 at 4 °C for 8 h, and purified using ultrafiltration (Amicon Ultra‐4, MWCO = 100 kDa) to remove the free pH‐M70. The obtained DoxFILN was stored in PBS buffer at 4 °C and used within 3 days. The amount of Dox was determined using LC‐MS. Whenever necessary, FITC‐labelled pH‐M70 was used to facilitate the quantification of M70 in the DoxFILN using a fluorescence spectrometer (F4600, HITACHI, Japan). The IL15c‐concentration of DoxFILN was evaluated by ELISA kit (Neobioscience Co., Ltd. Shenzhen, China) at different time point (0, 24, 48, and 72 h) under different pH (pH 6.5 and pH 7.4). DoxFILN^−M70^ was prepared with the same method without adding membrane‐bonded M70. FITC‐labelled Fn and Cy5‐labelled cell membranes were used for the preparation of FILN and FILN^−M70^ (a M70‐free version of FILN) for imaging purposes.

### Cy5‐Labeled Membrane

Cy5‐NHS ester (0.5 mg) in H_2_O (100 µL) was added dropwise to membrane solution (5 × 10^7^ cells, 500 µL) with 15 µL triethylamine and stirred overnight at 37 °C. The free Cy5 was removed using ultrafiltration (molecular weight cut‐off, 30 kDa).

### TEM

I‐pBMDC, Dox‐Fn, and DoxFILN were incubated in PBS, another solution of DoxFILN was adjusted to pH 6.5, 7 µL of each solution was added onto a copper grid and stained with 2% phosphotungstic acid. The morphology was observed on a Tecnai microscope (FEI, USA) at an accelerating voltage of 120 keV.

### Dynamic Light Scattering

The hydrodynamic sizes of the treated nanoparticles were measured by ZS90 (Malvern, UK). The samples were prepared by dispersing the nanoparticles in PBS (pH 7.4 or 6.5) for over 72 h at a concentration of 1 mg mL^−1^ (in BSA) at 25 °C.

### Drug Release in Vitro

Dox‐Fn, DoxFILN^−M70^ and DoxFILN were placed in buffers of pH 5.0, 6.4, or 7.4. The released Dox was separated from nanovesicles via ultracentrifugation (Himac CS150GXII, 10^5^ g, 4 °C). The amounts of Dox in the supernatants were quantified using LC‐MS. The FITC labeled Fn released from FILN and FILN^−M70^ in different pH were collected by centrifugation (CENCE H2050R, 2000 g, 4 °C) and the concentration of FITC‐Fn in the supernatant was quantified using a microplate reader (Enspire, PerkinElmer, Singapore).

### Cell Viability Assay

2 × 10^4^ 4T1 or CTLL‐2 cells were incubated in 8 mL 1640 medium at IL15c or IL15 concentration of 0.2 nm. The cells were stained with Trypan Blue (0.4%), and the density of the viable cells was determined using a cell counter (LifeTechnology) at 0, 24, 48, and 72 h. Calculated the fold change in cell density at each time point compared to 0 h. The experiment was performed in triplicates.

### Cellular Uptake In Vitro

4T1 cells (5 × 10^4^) and CTLL‐2 cells (5 × 10^4^) were seeded in 6‐well plates and co‐cultured overnight. The medium was replaced with fresh medium (pH 6.5) which containing FILN or FILN^−M70^. After 4 h incubation, adherent 4T1 cells and suspension CTLL‐2 cells were collected separately, the cellular uptake of the FITC‐labelled Fn and Cy5‐labelled membrane protein by 4T1 cells and CTLL cells were determined using flow cytometry.

### Subcellular Colocalization In Vitro and In Vivo

To investigate the subcellular colocalization of FILN and FILN^−M70^ within 4T1 cells and CTLL‐2 cells in vitro, 4T1 cells (5 × 10^4^) and CTLL‐2 cells (5 × 10^4^) were seeded in 30 mm Nunc glass‐bottom dish and co‐cultured overnight. The medium was replaced with fresh medium (pH 6.5) containing FILN or FILN^−M70^. After 4 h incubation, the suspension CTLL‐2 cells were collected and washed three times with PBS and transferred to a new 30 mm Nunc glass‐bottom dish. The adhesive 4T1 cells were also washed three times with PBS. Both of the cells were then imaged with confocal laser scanning microscopy (TCS SP8, Leica, Germany). For the in vivo experiment, tumor‐bearing mice were treated with fluorescence‐labelled FILN, FILN^−M70^, or Fn + I‐pBMDCN 8 h before harvesting the tumors. The tissues were fixed, sectioned, and stained with primary antibodies overnight at 4 °C, and then secondary antibodies. The slices were imaged using CLSM (TCS SP8, Leica, Germany).

### Biodistribution

The biodistribution of Dox in mice was quantified using LC‐MS. 4T1 tumor‐bearing BALB/c mice were treated with DoxFILN, DoxFILN^−M70^ or Dox‐Fn + I‐pBMDCN (Dox: 5 mg kg^−1^; IL15c: 85 pmole per mouse) as described above. At 2, 8, and 24 h after the injection, the major organs and tumors were collected and weighed. The intratumoral Dox was extracted with methanol and quantified by LC‐MS. The time‐dependent tumor exposure of Dox after the same treatment was further quantified using LC‐MS. The transitions m/z 544.4→m/z 397.0 of targeting molecule Dox was monitored and quantified. The data were normalized to the tissue weight and presented as mean ± s.e.m. Similarly, the mice were treated with Cy5 labeled FILN, FILN^−M70^ or Fn + I‐pBMDCN (IL15c: 85 pmole per mouse) as described above. At 2, 8, and 24 h after injection, the tumors were collected. The fluorescence from Cy5 labeled I‐pBMDC was imaged using an IVIS live animal imaging system (Xenogen, Alameda, CA). Cy5 labeled I‐pBMDCN was quantified using the semiquantitative method according to the fluorescence images.

### Pharmacokinetics

4T1 tumor‐bearing BALB/c mice were randomly grouped (*n* = 3) and injected intravenously with Cy5‐labeled DoxFILN, Dox‐Fn, or Cy5 labeled IL15 superagonist (Dox: 5 mg kg^−1^; Cy5: 0.1 mg kg^−1^). The blood samples were collected into EP tubes containing 10 µL of heparin sodium (10 mg mL^−1^) at different time points (15, 30 min, 1, 2, 4, 8, 12, 24, and 48 h). Then the Cy5 and Dox fluorescence intensities in blood were measured using a microplate reader. The pharmacokinetic parameters were analyzed by the DAS software.

### Immunogenic Cell Death In Vitro and In Vivo

4T1 cells were seeded into a 24‐well‐plate (4 × 10^4^ cells well^−1^) and cultured for 12 h. The cells were then incubated with PBS, DoxFILN, DoxFILN^−M70^ or Dox‐Fn + I‐pBMDCN (IL15c at 10 ng mL^−1^ equivalent) for 4 h and drug‐free medium for 12 h. For CRT quantification, the cells were sequentially stained with an anti‐CRT primary antibody and Alexa Fluor 647‐labeled rabbit anti‐mouse IgG before flow cytometry analysis. For HMGB1 imaging, the cells were fixed with 4% paraformaldehyde, permeabilized with 0.1% Triton X‐100, and stained with anti‐HMGB1 primary antibody, Alexa Fluor 488‐labeled rabbit anti‐mouse IgG and DAPI. The glass slides were washed with PBS thrice after each step and imaged on a laser scanning confocal microscopy. For ICD determination in vivo, orthotopic 4T1 tumor‐bearing mice (tumor volume ≈100 mm^3^) received PBS, DoxFILN, DoxFILN^−M70^ or Dox‐Fn + I‐pBMDCN (Dox: 5 mg kg^−1^; IL15c: 85 pmole per mouse, 3 injections), respectively. The mice were sacrificed by carbon dioxide asphyxiation, and the tumors were collected 3 days after the last injection (*n* = 5). The tumors were weighed, homogenized in PBS, and centrifuged to obtain the supernatant. For CRT and HMGB1 imaging in vivo, tumor slices were collected from mice receiving indicated treatments, and stained with fluorescence‐labelled antibodies against HMGB1 (green) and CRT (red) before visualization. The nucleus was stained with DAPI (blue).

### DC‐Mediated Costimulation of OT I Cells

DCs were collected from DLNs of mice bearing B16F10‐OVA tumors (≈200 mm^3^) that had undergone different treatments (PBS, DoxFILN, DoxFILN^−M70^ or Dox‐Fn + I‐pBMDCN). OT I cells were isolated from the spleen of OT I mice. To purify the cells, CD8a or CD11c positive selection was performed using MACS LS column with anti‐biotin microbeads following the manufacturer's protocol. OT I CD8^+^ T cells were labeled with CFSE (1 µm) following the manufacturer's introduction. 10000 DCs and 50000 CD8^+^ OT I cells were co‐cultured in RPMI 1640 medium containing 10% FBS, 1% antibiotics. Proliferation of T cells was determined by flow cytometry after a 48 h coincubation period.

### Antigen‐Specific T Cells

Mice bearing orthotopic B16F10‐OVA tumors (≈100 mm^3^) received 3 injections of DoxFILN, DoxFILN^−M70^, Dox‐Fn + I‐pBMDCN, or PBS (Dox: 5 mg kg^−1^; IL15c: 85 pmole per mouse). The tumors were collected 3 days after the last injection and homogenized to obtain a single‐cell suspension. The cells were stained with antibodies against CD45, CD3, and CD8, and tetramer against OVA_257‐264_‐H‐2Kb‐specific TCR. The percentages of tumor‐specific TILs (CD45^+^CD3^+^CD8^+^OVA_257‐264_‐H‐2Kb‐specific TCR^+^) among TILs (CD45^+^CD3^+^CD8^+^) were analyzed using flow cytometry.

### Cytokines Detection

Blood samples were harvested 3 days after the last injection and were stored for at least 6 h (4 °C). The samples were centrifuged to collect the serum. All the samples were frozen and stored at −80 °C if an analysis was not performed instantly. The levels of IL6, IL10, IFN‐γ, and TNF‐α in the serum were measured using ELISA kits according to the manufacturers’ protocols. Serum samples were also sent to Servicebio Technology Co., Ltd (Wuhan, China) for analysis of serum chemistry parameters. The data were presented as mean ± s.d.

### Tumor‐Infiltrating Lymphocytes

To validate the effect of DoxFILN in intratumoral immune cell recruitment and activation, mice bearing orthotopic 4T1 tumors were treated with intratumoral injection of DoxFILN, DoxFILN^−M70^, Dox‐Fn + I‐pBMDCN (Dox: 5 mg kg^−1^; IL15c: 85 pmole per mouse, 3 injections) or PBS. The tumors were collected 7 days after the last injection, weighted, cut into small blocks, and digested with a cocktail of enzymes at 37 °C for 90 min. The cells were filtered through nylon mesh, washed with fresh RMPI 1640 medium. The collected cells were stained with corresponding antibodies to determine the amounts of CD8^+^ T cells, active CD8^+^ T cells, NK cells, and active NK cells in the tumors. The data were presented as mean ± s.d.

### Tumor Growth Inhibition and Animal Survival Evaluation

Mice bearing orthotopic 4T1 or EMT6 tumors were randomly assigned into one of the 6 groups (*n* = 7), receiving DoxFILN, DoxFILN^−M70^, Dox‐Fn + I‐pBMDCN, Dox‐Fn, I‐pBMDCN or PBS at the same dosage (Dox: 5 mg kg^−1^; IL15c: 85 pmole per mouse, at day 0, day 4, and day 8). The tumor sizes were monitored every 2 days, and the tumor volume (*V*) was calculated from the major axis (*L*) and minor axis (*W*) of the tumors (*V* = (*L* × *W* ×* W*)/2). The mice were sacrificed when the tumor volume grew to 1500 mm^3^ for animal welfare.

### Statistical Analysis

Data were analyzed with a two‐tailed Student's *t*‐test when two groups were compared. One‐way or two‐way analysis of variance (ANOVA) and Tukey posthoc tests were used when more than two groups were compared (multiple comparisons). The survival benefit was determined using a log‐rank test. All statistical analyses were performed using the Prism software package (GraphPad Prism 6.0). Differences were considered to be statistically significant if *P* < 0.05.

## Conflict of Interest

The authors declare no conflict of interest.

## Supporting information



Supporting Information

## Data Availability

The data that support the findings of this study are available in the supplementary material of this article.
