# Supplementary material for: Interleukin 15‐Presenting Nanovesicles with Doxorubicin‐Loaded Ferritin Cores for Cancer Immunochemotherapy
Source: Adv Sci (Weinh). 2024 Dec 3;12(4):2409194. doi: 10.1002/advs.202409194 (PMC11789581; doi:10.1002/advs.202409194)
Supplement: Supplementary file 1 — Supporting Information [file ADVS-12-2409194-s001.docx]

© Copyright 2021. WILEY-VCH GmbH.

Supporting Information

Interleukin 15-Presenting Nanovesicles with Doxorubicin-Loaded Ferritin Cores for Cancer Immunochemotherapy

Yihui Zhai, Wen Zhang, Jinming Wang, Ying Kong, Rong Rong, Tianqun Lang, Chao Zheng, Yanke Wang, Yang Yu, Helen He Zhu, Ying Cai*, Pengcheng Zhang*, Yaping Li*

**Supporting Figures**


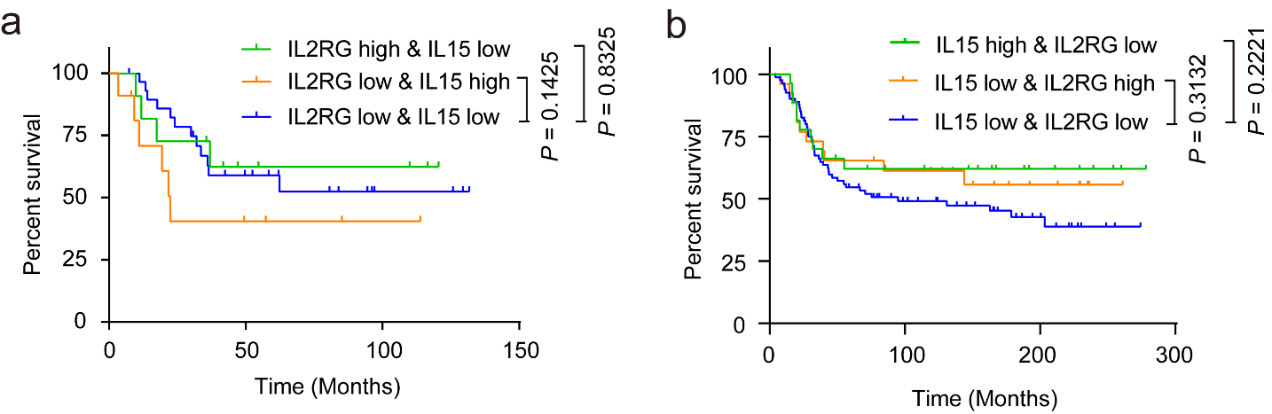


**Figure S1. Kaplan–Meier analysis of two cohorts TNBC patients.** Overall survival (OS) probability of TNBC patients from Sabatier cohort (n = 83 patients) (**a**) and METABRIC cohort (n = 216 patients) (**b**) with different transcription levels of intratumoral *IL2RG* and *IL15* genes. Statistical analysis was performed using two-sided log-rank test.


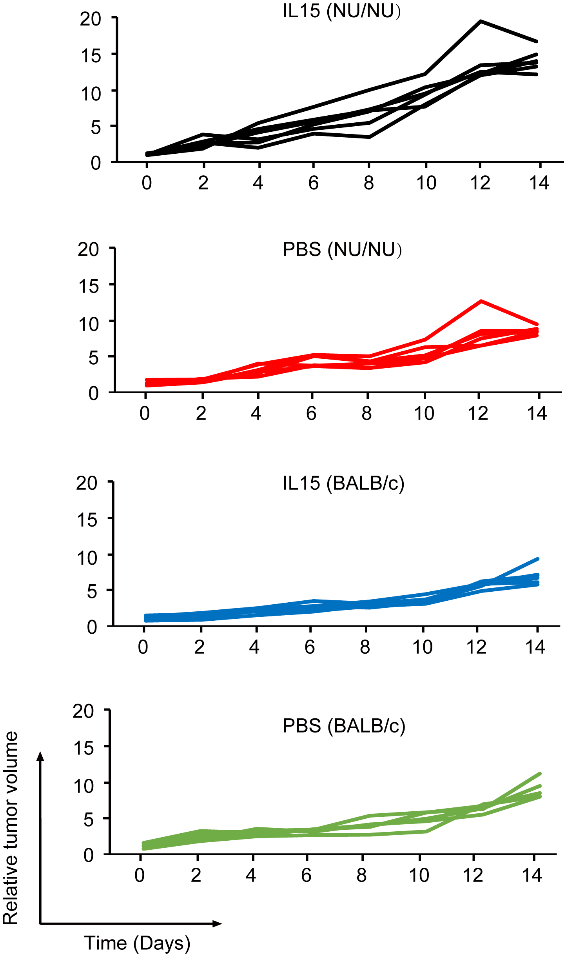


**Figure S2. Growth profiles of 4T1 tumors after IL15 treatment.** Individual tumor growth curves after the indicated treatments (PBS or IL15) in immunocompromised (NU/NU) and immunocompetent mice (BALB/c). (n = 6 biologically independent animals).


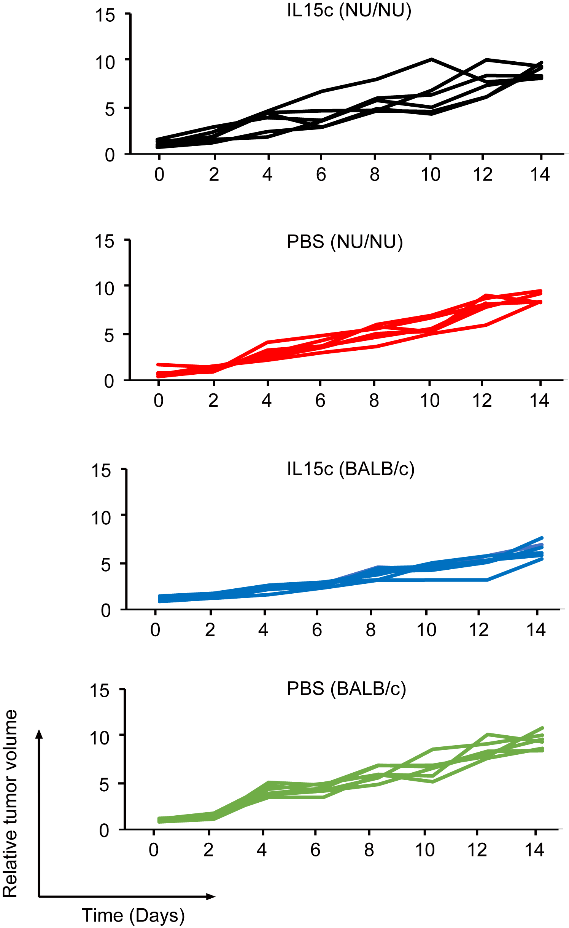


**Figure S3. Growth profiles of 4T1 tumors after IL15c treatment.** Individual tumor growth curves after the indicated treatments (PBS or IL15c) in immunocompromised (NU/NU) and immunocompetent mice (BALB/c). (n = 6 biologically independent animals).


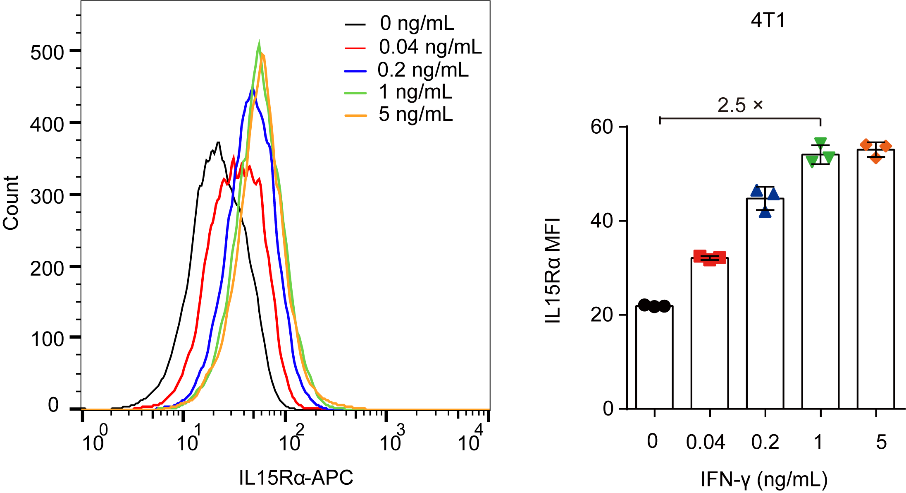


**Figure S4. Expression of IL15Rα on the surface of 4T1 after exposure to IFN-γ.** The 4T1 cancer cells were incubated with IFN-γ of a series of concentrations ranging from 0 to 5 ng mL^-1^ for 12 h. The surface IL15Rα level on the cancer cells was then determined using flow cytometry after staining with anti-IL15Rα antibodies.


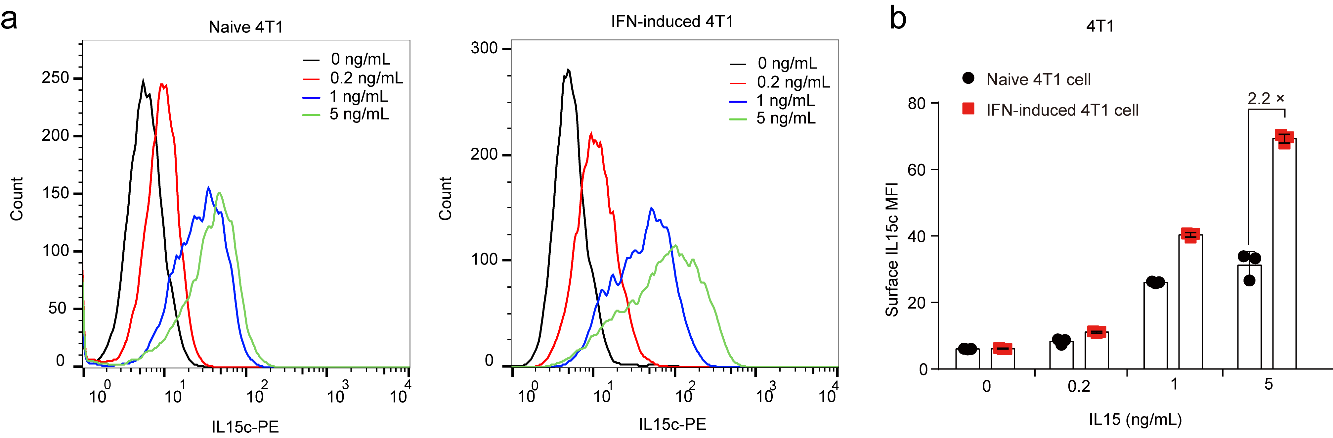


**Figure S5. The surface IL15c level of 4T1 cells after incubation with IL15.** Representative flow cytometry profiles (**a**) and quantification (**b**) of the surface IL15c. The naïve or IFN-γ-induced 4T1 cells were incubated with IL15 of a series of concentrations ranging from 0 to 5 ng mL^-1^ for 30 min. The IL15c were stained with anti-IL15c antibodies.


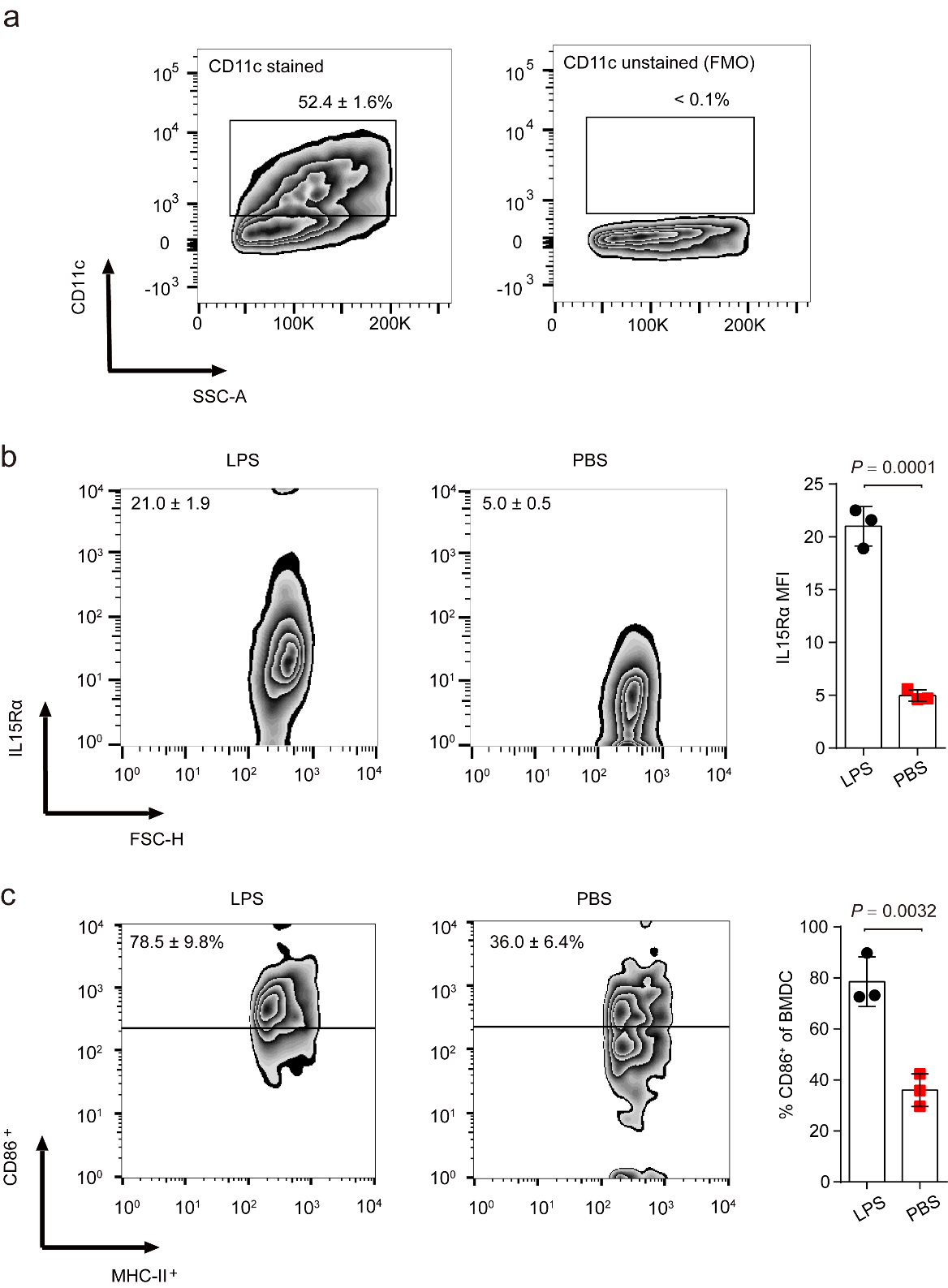


**Figure S6. Effect of LPS treatment on IL15Rα and CD86 expression on BMDC.** (**a**) The percentage of CD11c^+^ (DC) in the bone marrow-derived cells after 7 days induction with GM-CSF and IL2. The mean fluorescence intensity (MFI) of IL15Rα expressed on the BMDC (**b**) and the percentages of CD86^+^ BMDC (**c**) were analyzed using flow cytometry. All data were presented as mean ± s.d. (n = 3 biological replicates per group).


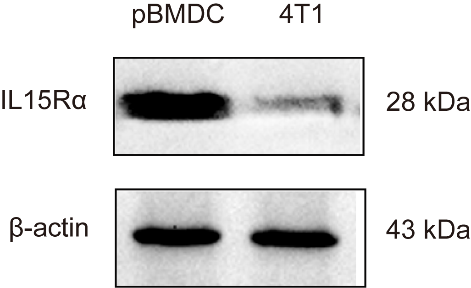


**Figure S7. Expression of IL15Rα on 4T1 and pBMDC.** Western blot analysis of IL15Rα and β-actin expression by 4T1 and pBMDC.


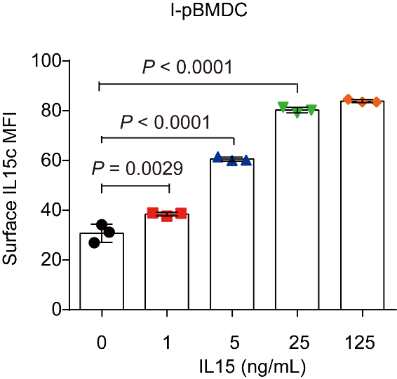


**Figure S8. The surface IL15c level of pBMDC after incubation with IL15.** The pBMDCs were incubated with IL15 of a series of concentrations ranging from 0 to 125 ng mL^-1^ for 30 min. The surface IL15c level on I-pBMDCs was then determined using flow cytometry after staining with anti-IL15c antibodies. Data were presented as mean ± s.d. (n = 3 biological replicates per group). Statistical significance was analyzed using one-way ANOVA and Tukey’s test.


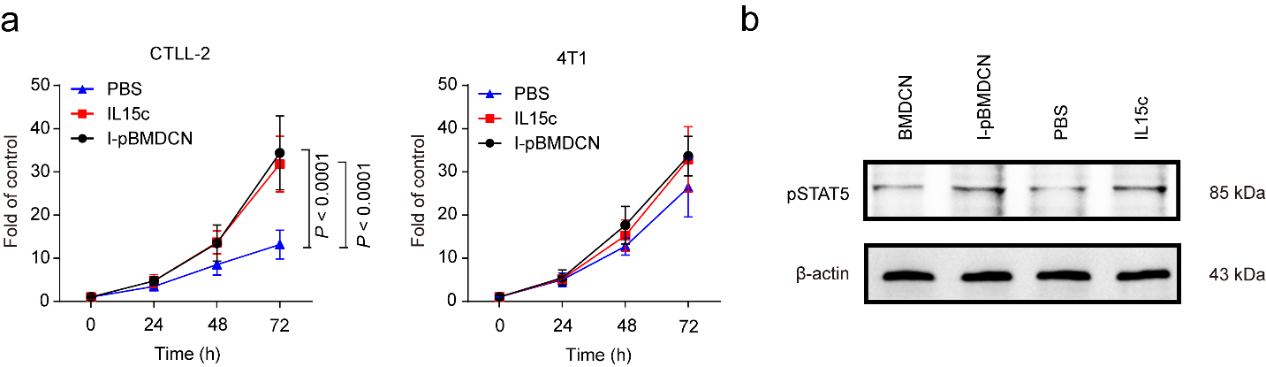


**Figure S9. Biological effect of I-pBMDCN.** (**a**) The growth profiles of CTLL-2 or 4T1 cells following the indicated treatments in 72 h. 4T1 or CTLL-2 cells (2×10^4^ cells) were incubated in 8 mL 1640 medium (IL15c of concentration of 0.2 nM equivalent). Data were presented as mean ± s.d. (n = 3 biological replicates per group). Statistical significance was analyzed by a two-way ANOVA test. (**b**) Western blot analysis of p-STAT5 and β-actin expression in CTLL-2 after treated by naïve BMDCN, I-pBMDCN, PBS or IL15c.


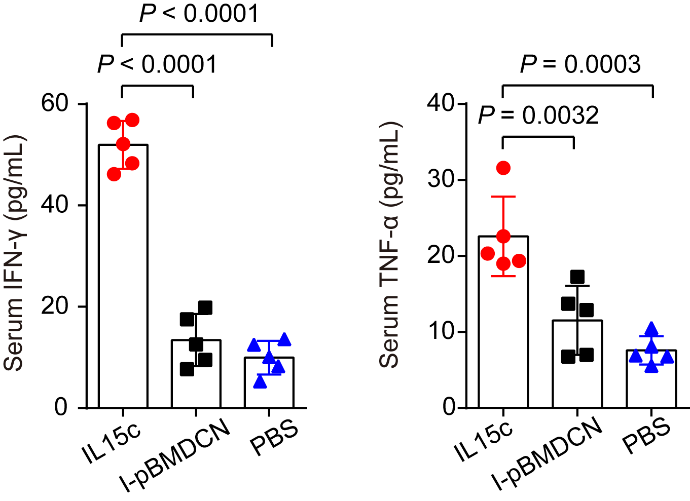


**Figure S10. Effect of IL15c, I-pBMDCN on animal serum IFN-γ and TNF-α.** Serum IFN-γ and TNF-α of mice were determined 3 days after the last injection of IL15c or I-pBMDCN after a 3-injection treatment regime (n = 5 biologically independent animals). Statistical significance was analyzed using one-way ANOVA and Tukey’s test.


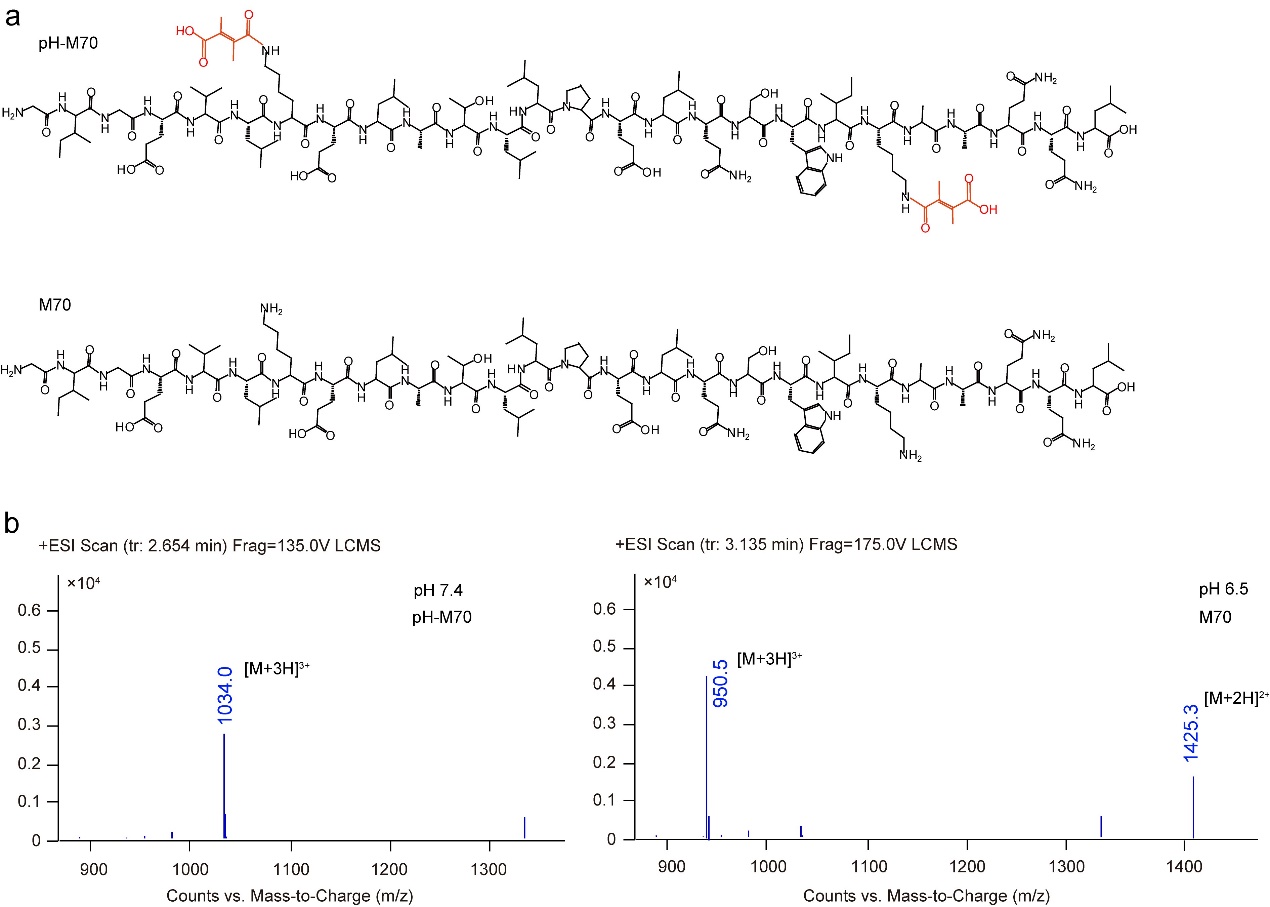


**Figure S11. Structure and characterization of caged M70 (pH-M70).** (**a**) Chemical structures of pH-M70 and M70. The membrane disruptive activity of M70 were caged *via* blocking the prime amine groups of the lysine residues. (**b**) Mass spectrum of pH-M70 at different pH. The estimated exact mass for pH-M70 was 3099.67 Da. The estimated exact mass for M70 was 2847.61 Da.


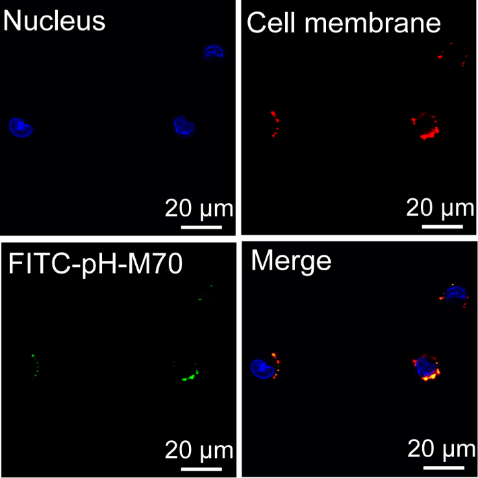


**Figure S12. Confocal images of FITC-pH-M70 decorated pBMDC.** pBMDCs (2 × 10^5^ cells/30 mm glass-bottom dish) were incubated with FITC-pH-M70 (10 µM, 25 ℃, 15 min), and then stained with DiI and Hoechst 33342 to visualize the cell membrane and nucleus.


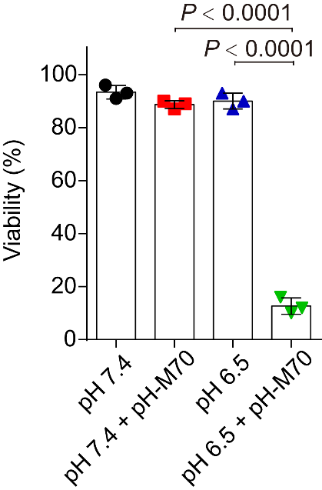


**Figure S13. Activation of membrane-bound pH-M70 in acid environment.** pBMDCs were incubated with pH-M70 in buffers of pH 6.5 or pH 7.4 in comparison with the control group. The cells were stained with Trypan Blue (0.4%) and the percentage of viable cells was determined using a cell counter (LifeTechnology). All data were presented as mean ± s.d. (n = 3 biological replicates per group). Statistical significance was analyzed using one-way ANOVA and Tukey’s test.


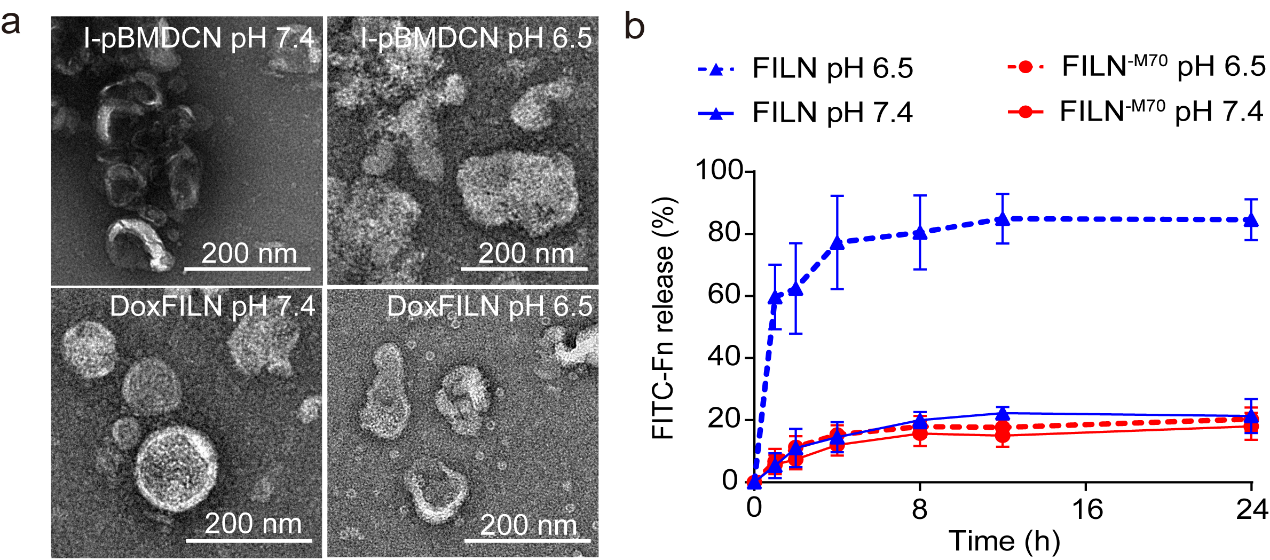


**Figure S14.** **The effect of M70 on the release of Dox-Fn from nanovesicles.** (**a**) Representative TEM images of I-pBMDCN and DoxFILN in pH 6.5 or pH 7.4. (**b**) The release profiles of FITC-labelled Fn from I-pBMDCN-coated Fn (FILN) and FILN^-M70^ in buffers of different pH. FILN^-M70^ is an FILN-like nanovesicle without membrane-bonded M70. The amounts of Fn were collected by centrifugal operation (3,000 rpm) and quantified using microplate reader (Enspire, PerkinElmer, Singapore). All data were presented as mean ± s.d. (n = 3 samples per group).


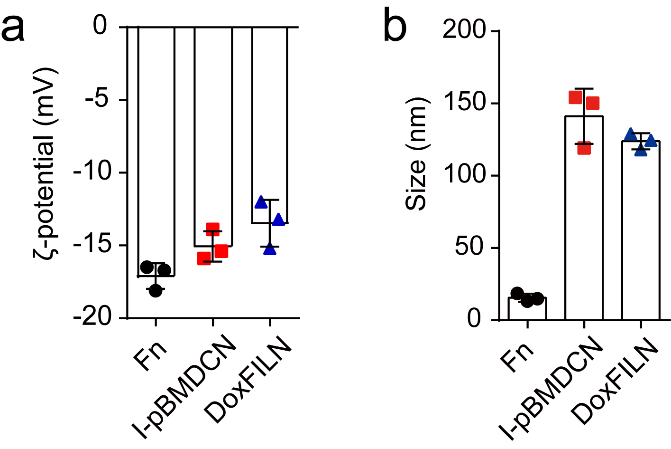


**Figure S15. Characterization of DoxFILN.** The ζ-potentials and hydrodynamic sizes of Dox-FN, I-pBMDCN and DoxFILN. All data were presented as mean ± s.d. (n = 3 samples per group).


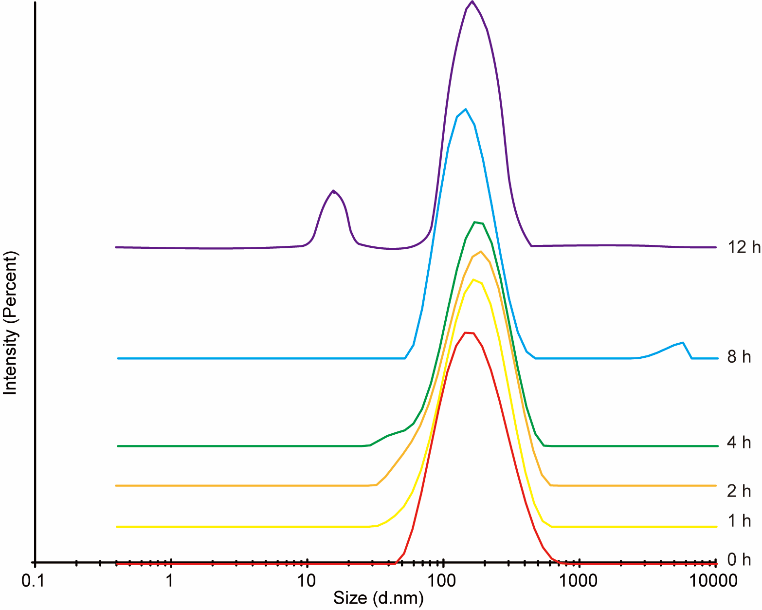


**Figure S16. Stability of DoxFILN.** The change of hydrodynamic sizes of DoxFILN at 37 ℃ in buffer of pH 7.4 under shaking conditions.


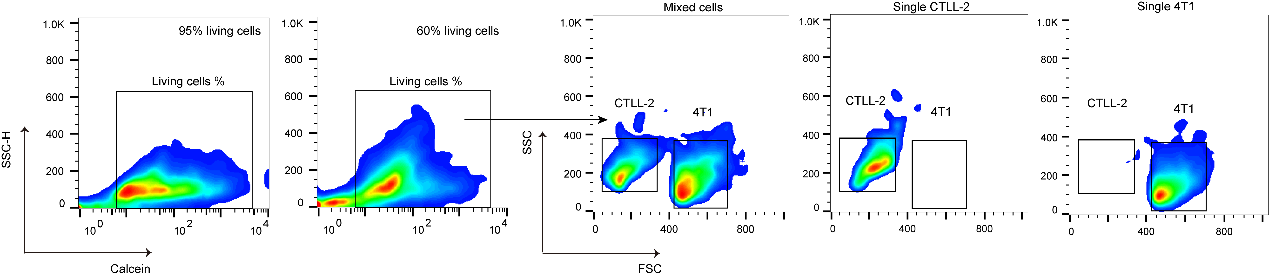


**Figure S17. Gating strategy for 4T1 and CTLL-2 cells in the co-cultures system.** First, calcei-positive living cells were gated. The mixed cells of CTLL-2 and 4T1 were then identified using different gates.


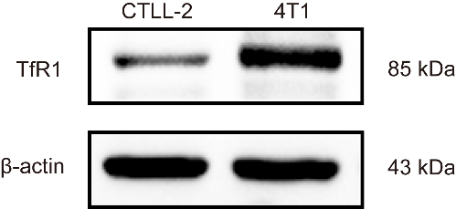


**Figure S18. Expression of TfR1 on 4T1 and CTLL-2.** Western blot analysis of TfR1 and β-actin expression in 4T1 and CTLL-2.


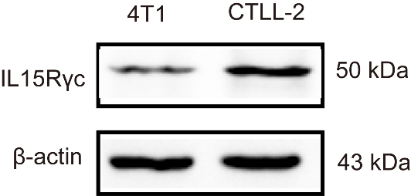


**Figure S19. Expression of IL15Rγc on 4T1 and CTLL-2 cells.** Western blot analysis of IL2RG and β-actin expression in 4T1 and CTLL-2.


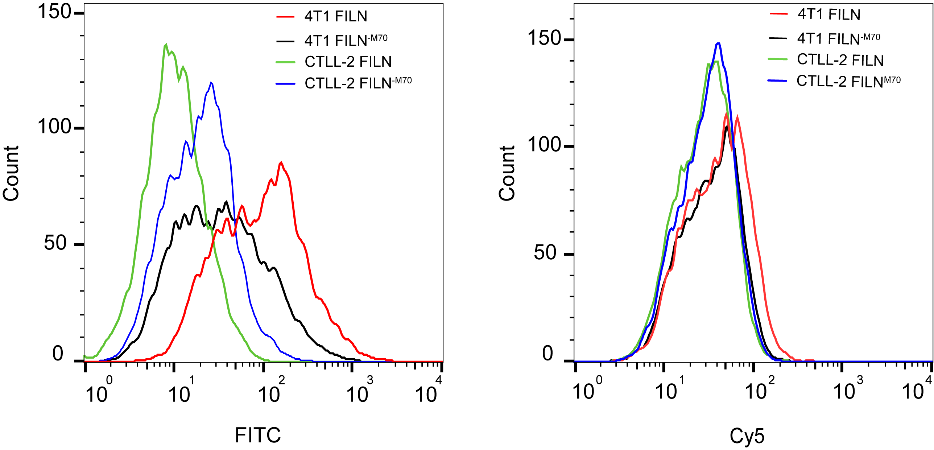


**Figure S20. The cellular uptake of FITC-labelled Fn or Cy5-labelled membrane protein in 4T1 or CTLL-2 cells.** The 4T1 cells were co-cultured with CTLL-2 cells. The FITC and Cy5 labelled FILN or FILN^-M70^ were added into the co-cultured system, after 4 h incubation, the uptake level of 4T1 cells and CTLL-2 cells were determined using flow cytometry.


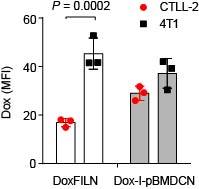


**Figure S21. Cellular uptake of nanoparticles.** Flow cytometry determination of the cellular uptake of Dox by 4T1 cells or CTLL-2 cells after a 4 h-incubation with DoxFILN or Dox-I-pBMDCN in buffer of pH 6.5. All data were presented as mean ± s.d. (n = 3 samples per group).


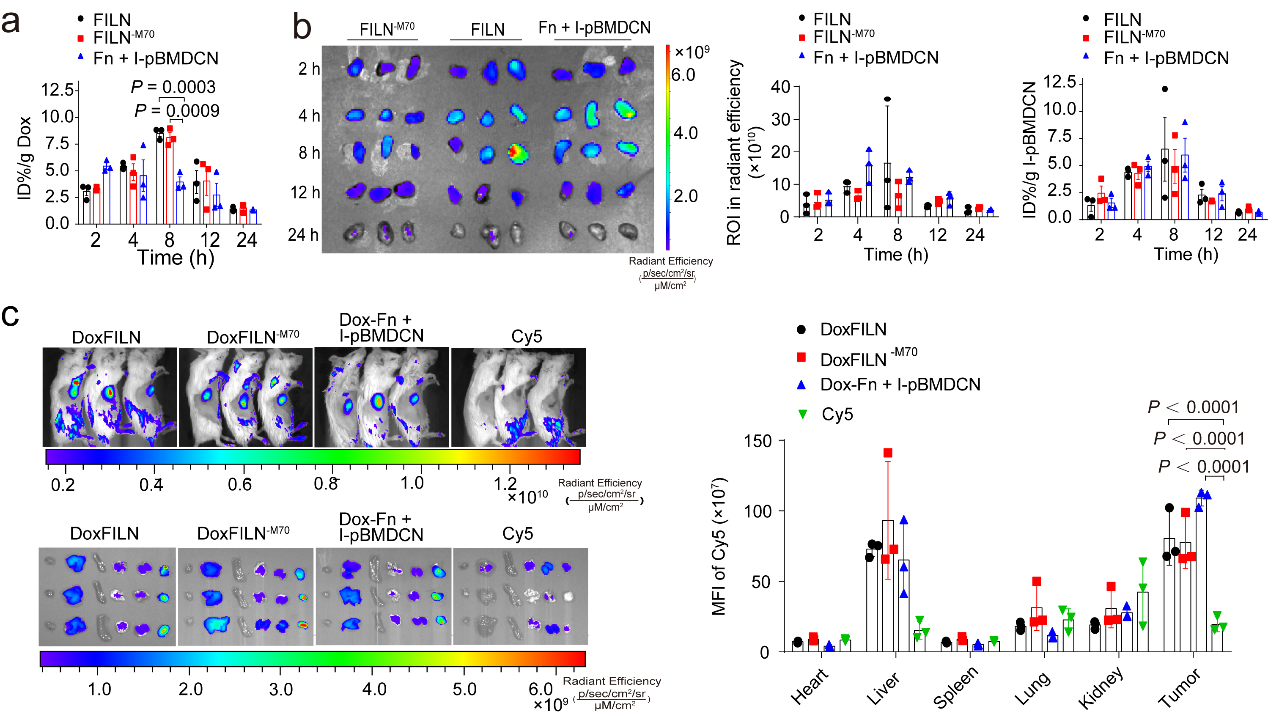


**Figure S22. Accumulation in the tumors.** The distribution of Dox (**a**) and I-pBMDCN (**b**) in tumor at 2 h, 4 h, 8 h, 12 h, and 24 h after injection of Cy5-labelled FILN, FILN^-M70^ and Fn + I-pBMDCN. Dox was quantified using LC-MS, and Cy5-labelled I-pBMDCN was semi-quantified based on the fluorescence intensity of ROI of the fluorescence images. (**c**) Fluorescence images and semi-quantification of Cy5 in the major organs and tumors of 4T1 tumor-bearing mice. All data were presented as mean ± s.d. (n = 3 biologically independent animals). Statistical significance was analyzed using one-way ANOVA and Tukey’s test.


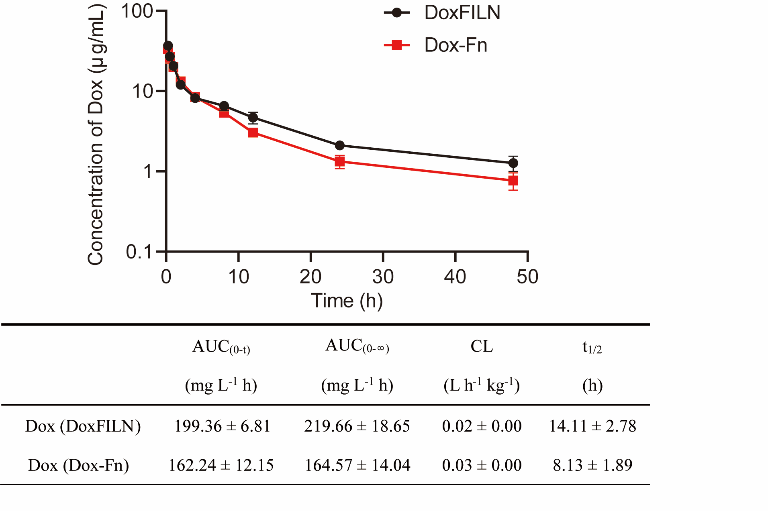


**Figure S23. The pharmacokinetics of Dox in vivo.** The blood concentration-time curve of Dox after intravenous administration of DoxFILN and Dox-Fn in mice (Dox: 5 mg kg^−1^). The Dox fluorescence intensity in blood was measured using a microplate reader. The pharmacokinetic parameters of the area under the curves (AUC), clearance (CL), and half-life (t_1/2_) of Dox in DoxFILN, and Dox-Fn were analyzed by the DAS software. Data were shown as mean ± SD (n = 3).


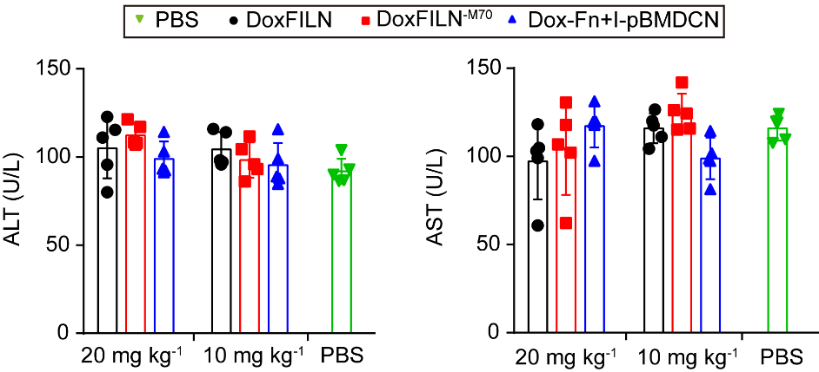


**Figure S24. Liver related blood biochemical index.** The concentrations of ALT and AST in the serum collected from mice receiving the indicated treatments on day 5 after one injection. All data were presented as mean ± s.d. (n = 5 biologically independent animals).


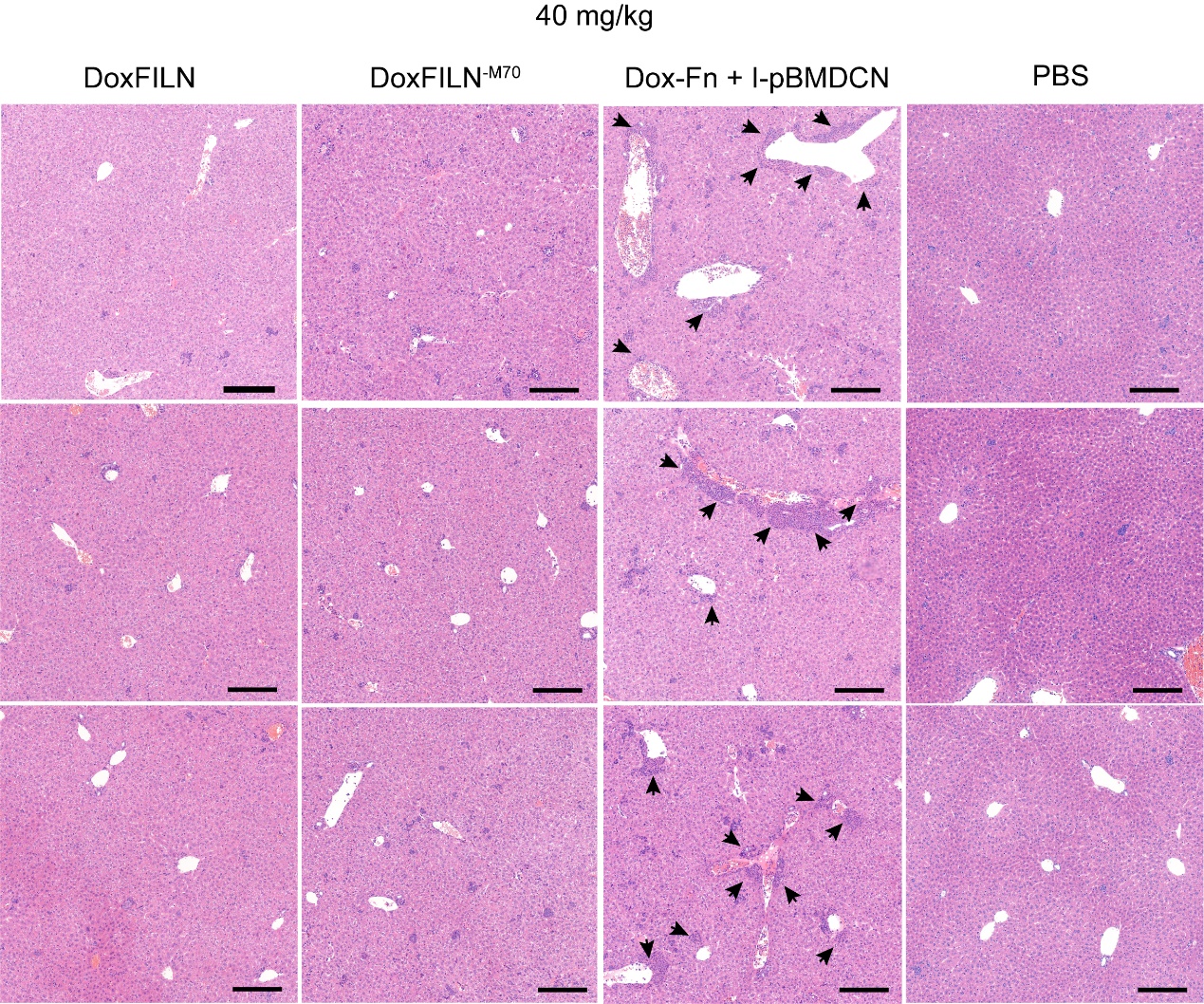


**Figure S25. The H&E results of livers.** Mice bearing orthotopic 4T1 tumors received 1 injection of the indicated treatments at dose of 40 mg/kg (Dox). After 5 days, the livers were collected and sliced, the arrows showed us visible immune cell aggregation in the livers of Dox-Fn + I-pBMDCN-treated mice. Scale bar = 200 μm.


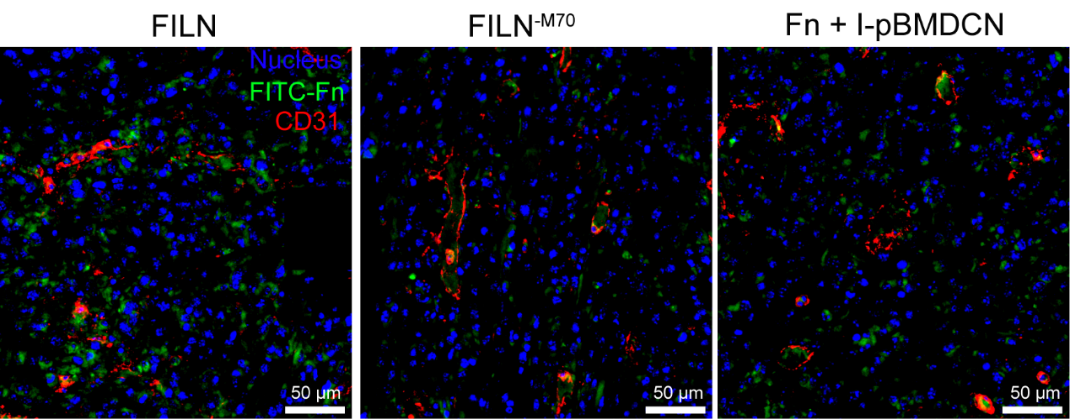


**Figure S26. Tumor distribution of Dox-Fn.** Fluorescence images of tumor slices collected from mice receiving indicated treatments at 8 h after the injection. The Fn was labelled with FITC, the nucleus was stained with DAPI (blue) and the tumor vessel was stained with CD31 (red).


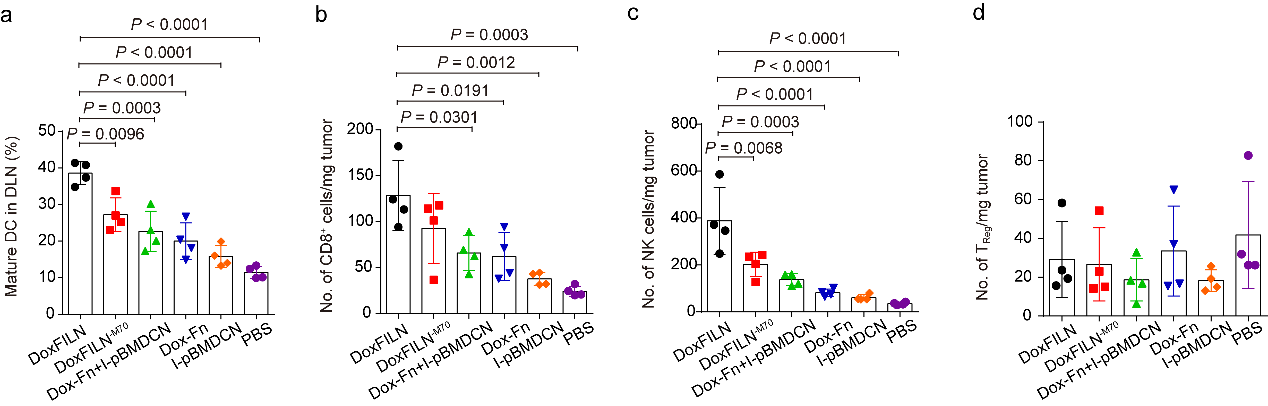


**Figure S27. The percentage of mature DCs (CD80^+^CD86^+^) in the draining lymph nodes and numbers of tumor-infiltrating immune cells.** Mice bearing orthotopic 4T1 tumors received 3 injections of the indicated treatments. (**a**) Three days after the last injection, the draining lymph nodes were collected to prepare a single-cell suspension. The cells were stained with antibodies against CD11c, MHC-II, CD80, and CD86, and analyzed using flow cytometry. (**b-d**) The tumors were collected 7 days after the last injection and homogenized to obtain a single-cell suspension. The cells were stained with antibodies against CD3, CD4, CD8 and CD49b. The number of CD3^+^CD8^+^ T cells (**b**), NK cells (**c**) and T_Reg_ (**d**) were quantified using flow cytometry. All data were presented as mean ± s.d. (n = 4 biologically independent animals). Statistical significance was analyzed using one-way ANOVA and Tukey’s test.


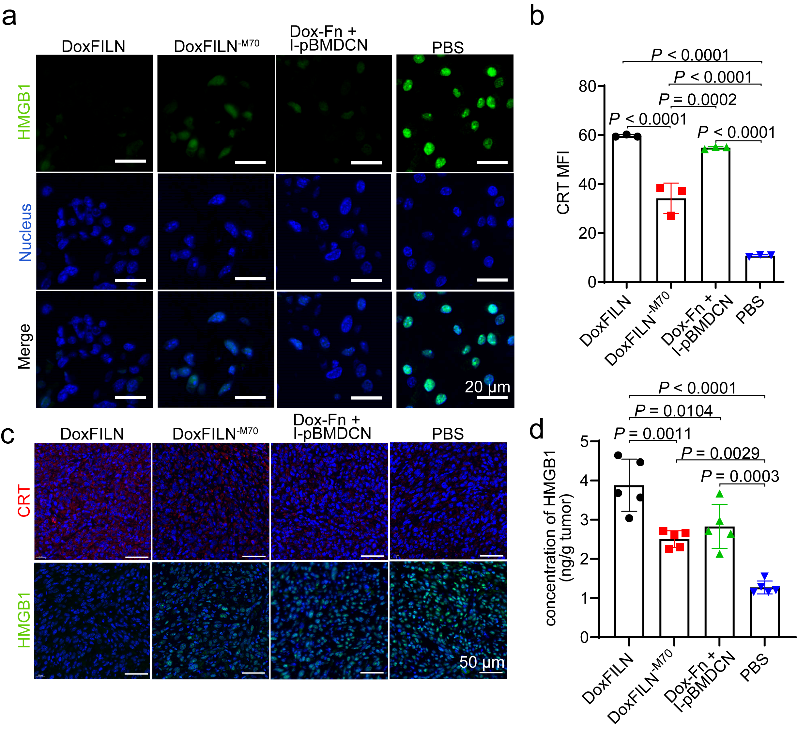


**Figure S28. The ICD of 4T1 in vitro and vivo.** (**a**) Confocal images of 4T1 cells treatment with DoxFILN, DoxFILN^-M70^ and Dox-Fn + I-pBMDCN. The nucleus and HMGB1 were stained with DAPI and anti-HMGB1 antibody- Alexa Fluor 488, respectively. Scale bar = 20 μm. (**b**) Quantification of CRT on cell membranes by flow cytometry. (**c**) Mice bearing orthotopic 4T1 tumors received 3 injections of the indicated treatments. The tumors were collected 3 days after the last injection. Fluorescence images of tumor tissues stained with DAPI, anti-HMGB1 antibody-Alexa Fluor 488, and anti-CRT antibody-Alexa Fluor 647. Scale bar = 50 μm. (**d**) Quantification of extracellular HMGB1 in vivo by ELISA kit. All data were presented as the mean ± s.d. (n = 3 independent samples in vitro; n = 5 biologically independent animals in vivo). Statistical significance was analyzed using one-way ANOVA and Tukey’s test.


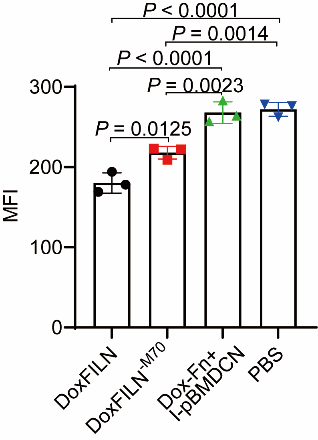


**Figure S29. DC-mediated costimulation of OT I cells.** MFI of CFSE-labelled OT I cells when co-incubated with DCs harvested from tumors receiving DoxFILN, DoxFILN^-M70^, Dox-Fn + I-pBMDCN, or PBS. The data were presented as mean ± s.d. (n = 3 biologically independent samples). Statistical significance was analyzed using one-way ANOVA and Tukey’s test.


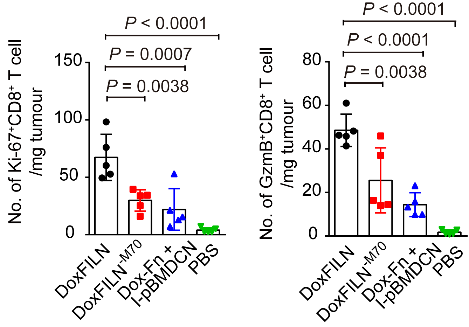


**Figure S30. Densities of Ki-67^+^ and GzmB^+^ cytotoxic T lymphocytes *in vivo*.** Mice bearing orthotopic 4T1 tumors received 3 injections of the indicated treatments. The tumors were collected 7 days after the last injection and homogenized to obtain a single-cell suspension. The cells were fixed, permeabilized, and stained with antibodies against CD3, CD8, Ki-67/GzmB. The percentages of proliferating (CD3^+^CD8^+^Ki-67^+^) and active (CD3^+^CD8^+^GzmB^+^) T cells among tumor-infiltrating cytotoxic T lymphocytes (CD3^+^CD8^+^) were analyzed using flow cytometry. All data were presented as mean ± s.d. (n = 5 biologically independent animals).


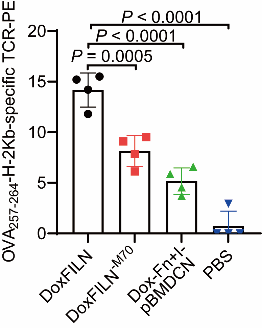


**Figure S31. Upregulation of OVA_257-264_-H-2Kb specific TCR on T cells.** Mice bearing orthotopic B16F10-OVA tumors received 3 injections of the indicated treatments. The tumors were collected 3 days after the last injection and homogenized to obtain a single-cell suspension. The cells were stained with antibodies against CD45, CD3, and CD8, and with tetramer for OVA_257-264_-H-2Kb-specific TCR. The percentages of tumor-specific TILs (CD45^+^CD3^+^CD8^+^OVA_257-264_-H-2Kb-specific TCR^+^) among TILs (CD45^+^CD3^+^CD8^+^) were analyzed using flow cytometry. All data were presented as mean ± s.d. (n = 4 biologically independent animals). Statistical significance was analyzed using one-way ANOVA and Tukey’s test.


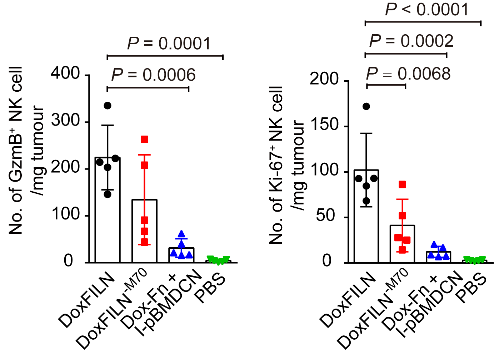


**Figure S32. Densities of Ki-67^+^ and GzmB^+^ NK cells *in vivo*.** Mice bearing orthotopic 4T1 tumors received 3 injections of the indicated treatments. The tumors were collected 7 days after the last injection and homogenized to obtain a single-cell suspension. The cells were fixed, permeabilized, and stained with antibodies against CD3, CD49b, Ki-67/GzmB. The percentages of proliferating (CD3^-^CD49b^+^Ki-67^+^) and active (CD3^-^CD49b^+^GzmB^+^) cells among tumor-infiltrating NK cells (CD3^-^CD49b^+^) were analyzed using flow cytometry. All data were presented as mean ± s.d. (n = 5 biologically independent animals).


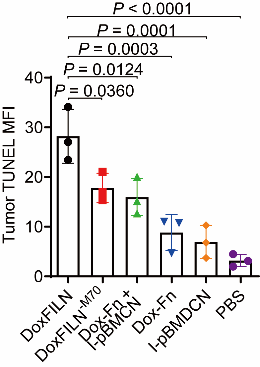


**Figure S33.** **Pro-apoptotic efficacy of DoxFILN in vivo.** Semi-quantitative determination of 4T1 cell apoptosis according to TUNEL fluorescence images of tumors collected at day 14. Data were analyzed by ImageJ. All data were presented as mean ± s.d. (n = 3 samples per group). Statistical significance was analyzed using one-way ANOVA and Tukey’s test.


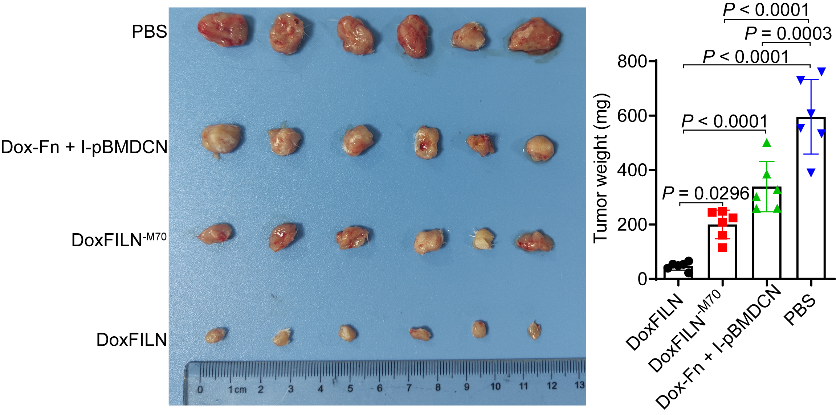


**Figure S34. Anti-tumor efficacy of DoxFILN in vivo.** Mice bearing orthotopic 4T1 tumors received 3 injections of DoxFILN, DoxFILN^-M70^, Dox-Fn + I-pBMDCN, or PBS (Dox: 5 mg kg^-1^; IL15c: 85 pmole per mouse). The tumors were collected from mice after 14 days. Data were presented as mean ± s.d. (n = 6 biologically independent animals). Statistical significance was analyzed using one-way ANOVA and Tukey’s test.


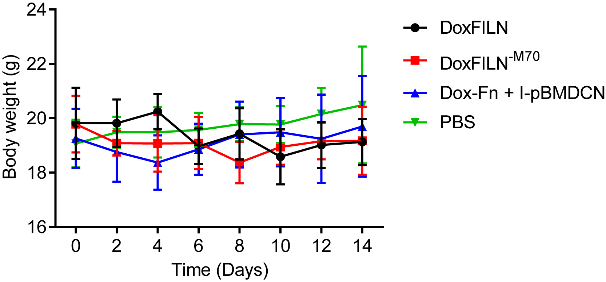


**Figure S35. Body weight change in mice.** Mice bearing orthotopic 4T1 tumors received 3 injections of DoxFILN, DoxFILN^-M70^, Dox-Fn + I-pBMDCN, or PBS (Dox: 5 mg kg^-1^; IL15c: 85 pmole per mouse). Mice weights were measured every two days. Data were presented as mean ± s.d. (n = 7 biologically independent animals).


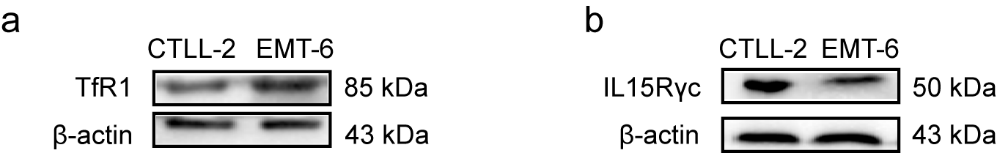


**Figure S36. Expression of TfR1 and IL15Rγc on EMT-6 and CTLL-2.** Western blot analysis of TfR1 (**a**), IL15Rγc (**b**) expression on EMT-6 and CTLL-2.


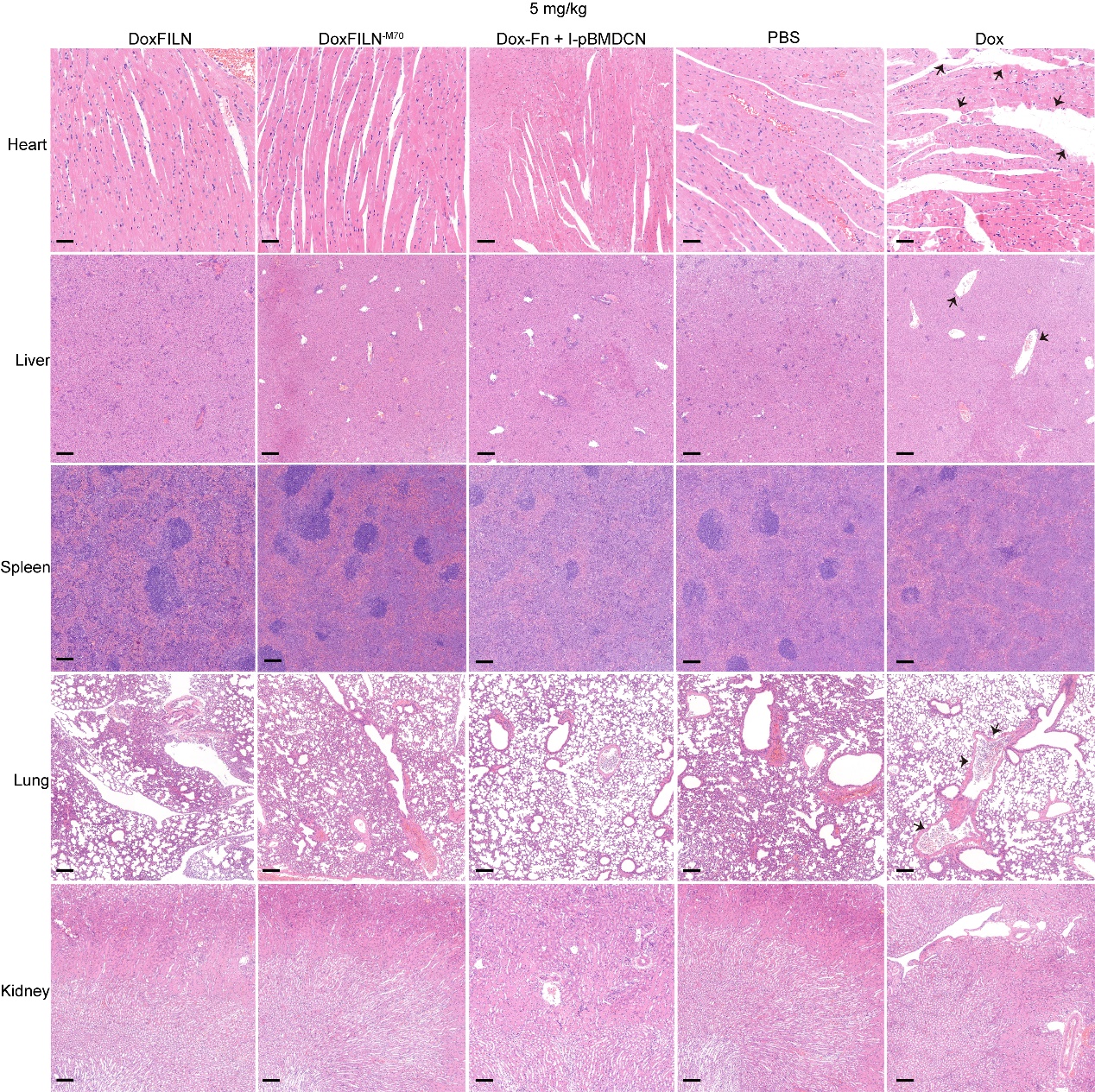


**Figure S37. The H&E results of different Organs.** Mice bearing orthotopic 4T1 tumors received 3 times i.v. injection, day 0, 4, 8 of the indicated treatments at dose of 5 mg/kg (Dox). On the sixth day after the last injection, the organs were collected and sliced. Scale bar = 200 μm.


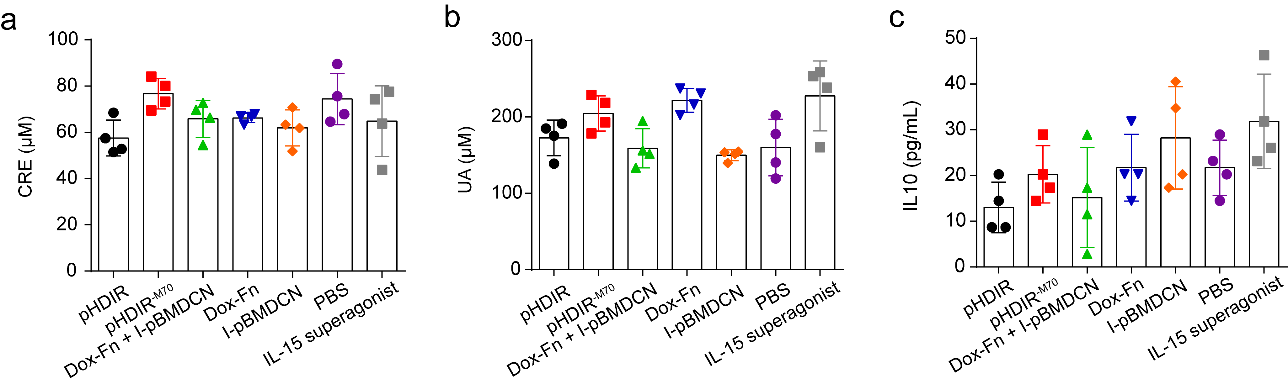


**Figure S38**. **Blood chemistry parameters and IL10.** (**a, b**) The concentrations of CREA (**a**) and UREA (**b**) in the serum collected from mice receiving the indicated treatments on day 3 after the third injection. (**c**) The concentrations of IL10 in the serum after 3 injections of the indicated treatments. All data were presented as mean ± s.d. (n = 4 biologically independent animals).


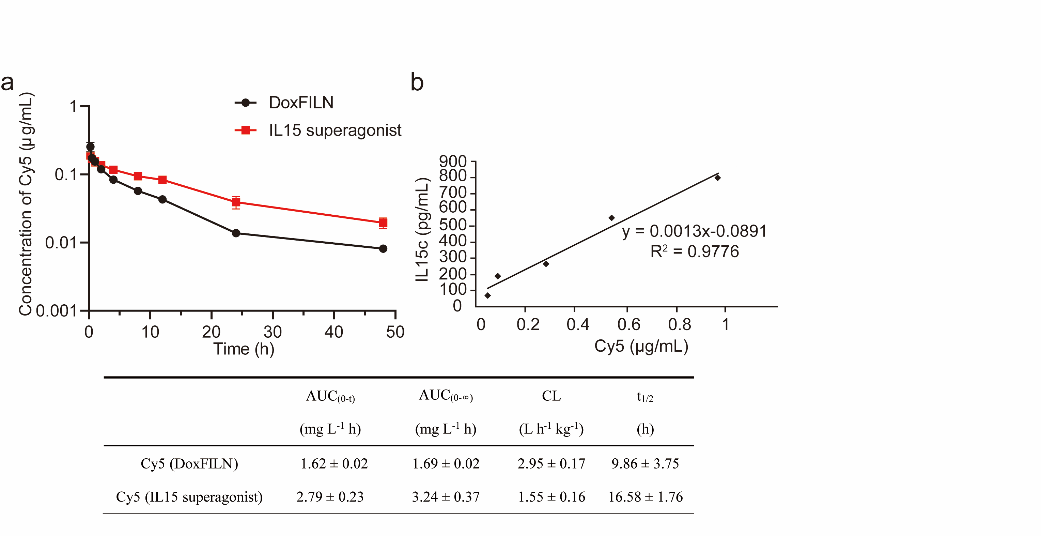


**Figure S39. The pharmacokinetics of IL15c in vivo.** (**a**) The blood concentration-time curve of Cy5 after intravenous administration of Cy5-labeled DoxFILN, and Cy5-labeled IL15 superagonist in mice (Cy5: 0.1 mg kg^−1^). The Cy5 fluorescence intensity in blood was measured using a microplate reader. The pharmacokinetic parameters of the area under the curves (AUC), clearance (CL), and half-life (t_1/2_) of Cy5 in DoxFILN, and IL15 superagonist were analyzed by the DAS software. Data were shown as mean ± SD (n = 3). (**b**) The concentration of IL15c was detected by ELISA kit in vitro, and the fluorescence intensity of Cy5 was positively correlated with the fluorescence intensity of Cy5-labeled IL15c.


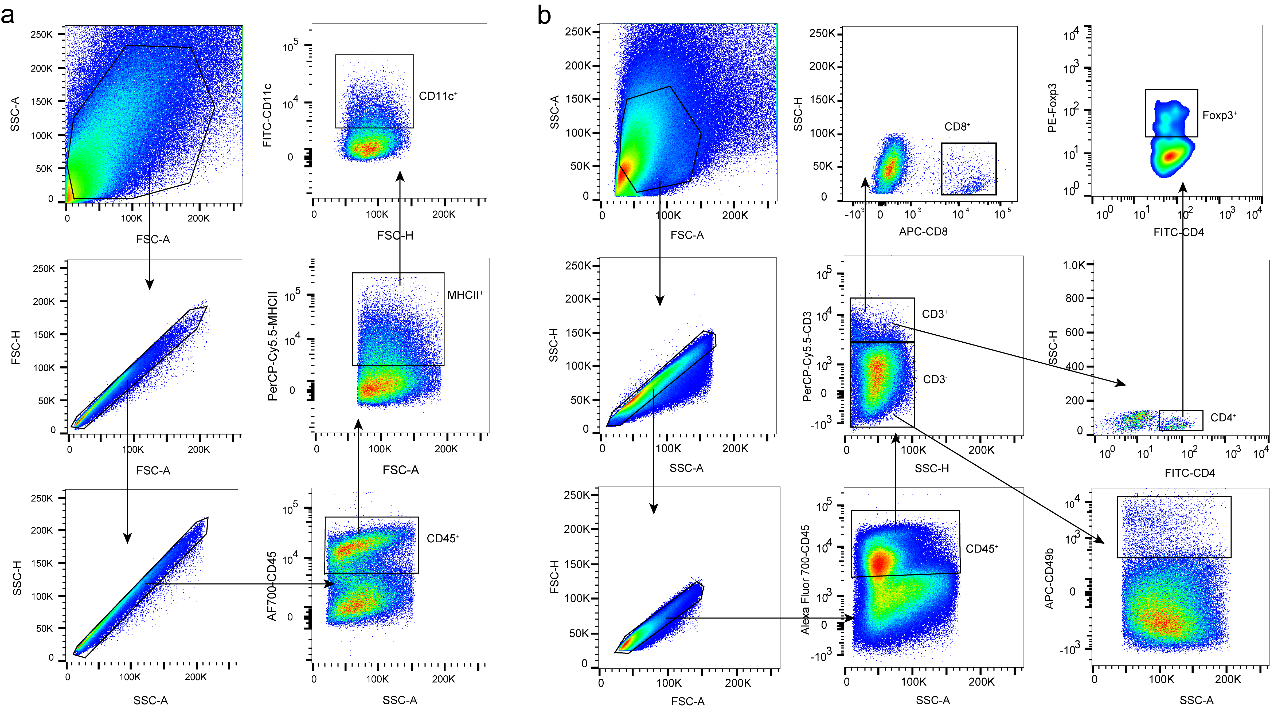


**Figure S40**. **Gating strategies for immune cells.** Herein, a sequential gating strategy was performed to exclude doublets. First SSC-H was plotted vs. SSC-A and then FSC-H vs. FSC-A for singlet population. (**a,b**) CD45^+^ cells were then gated for immune cells. (**a**) Gating strategy for intratumoral DCs (CD11c^+^MHC-II^+^). (**b**) Gating strategy for intratumoral CD4^+^ T cells (CD3^+^CD4^+^), CD8^+^ T cells (CD3^+^CD8^+^) and NK cells (CD3^-^CD49b^+^).
